# Pollucite Ceramics and Glass-Ceramics as Advanced Wasteforms for the Immobilization of Cs-Loaded IONSIV Wastes

**DOI:** 10.1021/acs.est.5c00266

**Published:** 2025-04-15

**Authors:** Ghazaleh Bahmanrokh, Edward Whitelock, Pranesh Dayal, Robert D. Aughterson, Anton Peristyy, Phillip Sutton, Rifat Farzana, Joel L. Abraham, Jess Degeling, Michael Page, Charles C. Sorrell, Pramod Koshy, Daniel J. Gregg

**Affiliations:** † Australian Nuclear Science and Technology Organisation, Locked Bag 2001, Kirrawee DC, New South Wales 2232, Australia; ‡ School of Materials Science and Engineering, UNSW Sydney, Sydney, New South Wales 2052, Australia

**Keywords:** nuclear waste, cesium immobilization, ion-exchange, pollucite glass-ceramic wasteform, chemical and mechanical durability, waste loading

## Abstract

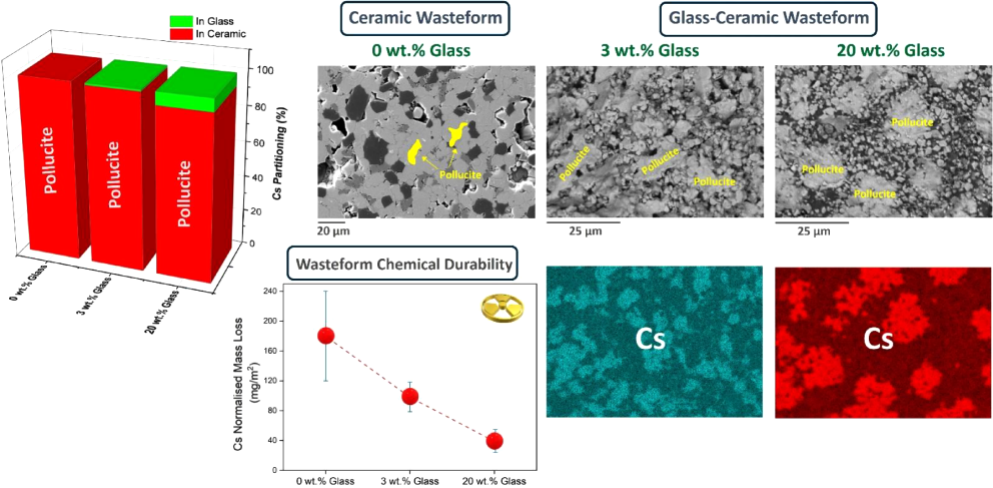

IONSIV R9120-B is a commercial inorganic ion exchange material used in the nuclear industry for the removal of Cs-137 from contaminated liquids. Once IONSIV is loaded with radioactive species, it is considered waste and requires treatment by incorporation into a stable wasteform to prevent radionuclide release during disposal. This work presents a promising, novel candidate glass-ceramic wasteform based on pollucite for the immobilization of cesium-loaded IONSIV. The tailored glass-ceramic provides chemical and processing flexibility through the addition of small amounts of glass formers, with cesium partitioned predominantly to the more chemically durable ceramic phase. A high waste loading of ∼70–80 wt % was achieved, along with a consistent phase assemblage of pollucite, srilankite, rutile, and glass. The chemical durability of the candidate wasteform was assessed using the ASTM C1285 standard method, with the results indicating high chemical durability relative to other candidate materials for cesium immobilization. A single preferred pollucite glass-ceramic design with a 70 wt % waste loading was selected and validated using unloaded and Cs-loaded IONSIV. Importantly, the design ensures consistent phase formation irrespective of Cs-loading on IONSIV, demonstrating tolerance to accommodate compositional variations in the waste.

## Abstract 2

This study provides a novel solution to prevent radioactive Cs release into the environment following its immobilization in a glass-ceramic.

## 1 Introduction

Ion exchange materials are employed in the nuclear industry for the removal of radioactive contaminants from water systems at nuclear facilities, such as nuclear power plants, fuel reprocessing plants, nuclear research centers, and liquid radioactive waste management systems.^1^ Crystalline silico-titanate (CST),^2−5^ zeolites,^6^ and ammonium phosphomolybdates^7^ are examples of microporous inorganic absorbents that are used in the industry for the removal of cesium (e.g., Cs-137) and strontium (e.g., Sr-90) radioactive isotopes, or to reduce their concentration in the liquid to allow its reclassification, for example, from high-level waste (HLW) to low-level waste (LLW).^8,9^

The commercial material IONSIV R9120-B (generic formula of (Na_2–*x*_)(Nb_*x*_Ti_2–*x*_)O_3_SiO_4_.2H_2_O) is a zirconium hydroxide (Zr(OH)_4_)-bound form of a CST ion exchanger^10^ with significant promise for nuclear waste applications.^11,12^ IONSIV is a modified version of sitinakite with cubane-like [Ti_4_O_4_]^8+^ clusters formed by four edge-sharing TiO_6_ octahedra,^13^ where cubane-like [Ti_4_O_4_]^8+^ clusters are connected by sharing vertices with SiO_4_ tetrahedra to form a three-dimensional system of crossed channels and a porous, polymeric solid structure. IONSIV is contacted with liquid radioactive waste streams via columns, where Cs^+^ ions from contacting solutions enter the porous structure and exchange intracrystalline positions with Na^+^ with high selectivity over a wide pH range.^14−16^ Ion exchange materials have been used to remove Cs from HLW at U.S. Department of Energy sites, for example, the Savannah River Site.^17−20^ IONSIV was also utilized in the Simplified Active Water Retrieve and Recovery System (SARRY) to remove Cs and Sr from contaminated water at the Fukushima Daiichi Nuclear Power Plant in Japan.^21,22^

As it is not practical to recycle CST once loaded with radioactive species, it is considered waste and requires treatment by incorporation into a stable matrix or “wasteform”. The wasteform provides the required structural stability and minimizes radionuclide release following disposal. Though Cs-loaded IONSIV is considered intermediate-level waste (ILW) in many countries,^23^ there is currently no disposal path for the material; thus, it is currently held in safe interim storage on several nuclear sites. Wasteform options under consideration for radioactively contaminated CST include incorporation into cementitious forms,^23^ vitrification into glass hosts,^24,25^ immobilization into a ceramic form,^24,26^ or recently, immobilizing the CST within specialized glass-ceramic wasteforms.^27−29^

Cement has been utilized for the solidification of LLW and, in some cases, for ILW.^23^ With regard to IONSIV, however, Cs can undergo ion exchange with sodium and potassium within the cement composition and leach into the surrounding environment through the cement pore water.^23,30^ Further, cement has a low waste loading^31^ and, therefore, produces large waste volumes for disposal.

A glass wasteform was investigated for the immobilization of CST following the treatment of highly alkaline liquid waste from the Melton Valley Storage Tanks at Oak Ridge, USA.^32−34^ Aqueous durability assessments using the ASTM C1285 standard test method^35^ indicated that acceptable glass wasteforms (more durable than the Environmental Assessment (EA) reference glass^36^) could be produced with a CST waste loading of 29 wt %.^33^

Ceramic materials produced by sintering or hot-uniaxial/hot-isostatic pressing (HUP/HIP) are potential candidate wasteforms due to the very high chemical durability of some mineral phases.^37−40^ A number of ceramic phases have been studied for the immobilization of Cs, including hollandite,^41−44^ pollucite,^45−47^ sodium zirconium phosphate,^48−50^ and apatite^51^ due to their Cs incorporation capacity and their chemical and mechanical durability. However, Cs is only a very small chemical component of the Cs-loaded IONSIV waste (predominantly Si, Ti, Nb, and Na in various quantities), and therefore, additional phase(s) are required to immobilize the entirety of the waste chemistry. The effect of the HIPing CST material without glass or ceramic-forming additives (i.e., ∼100 wt % waste loading) was considered previously.^27^ A multiphase ceramic was produced containing several Cs-bearing phases that varied depending on the Cs loading. Such phases included Cs_2_TiNb_6_O_18_, Cs_2_ZrSi_6_O_15_, and Cs_2_ZrSi_3_O_9_, and though hollandite was a target phase, it was not formed.

Glass-ceramics (GCs) are composite materials that combine the advantages of both glass and ceramic materials. The nuclear wasteform community defines several types of product materials as GCs and classifies them according to the product itself or the process that produces them.^52^ GCs are chemically more flexible and easier to process than pure ceramics, but they also possess the enhanced chemical durability of ceramics relative to conventional borosilicate glasses.^53^ To form GCs, ceramic phases are crystallized by design within a glass matrix. Effective GC compositions have been densified *via* sintering;^24^ however, HIPing is a consolidation method that has been shown to improve the chemical durability and densification of the material, reduce the final volume, and avoid the volatilization of species like Cs. Most importantly, GCs can provide an increased tolerance to variation in waste composition, which may arise from the varying rate of Cs incorporation in the IONSIV. The target Cs-bearing mineral phase within the GC is pollucite (CsAlSi_2_O_6_), a promising material for Cs-bearing waste immobilization owing to its relatively high chemical durability and high potential Cs incorporation (>40 wt %).^54−56^ Pollucite has a cubic (space group *I*41/*a*) or tetragonal (space group *Ia*3*d*) structure,^57^ with the general formula (Na, K, Rb, Cs)MgAl_0.5_P_1.5_O_6_, and is known to incorporate a vast range of cations.^56^ Though pollucite has been found to have lower chemical durability (0.093 g m^–2^ d^–1^ for 28 days, ASTM C1220 test^47^) relative to some other Cs-bearing mineral phases, for example, hollandite (0.003–0.02 g m^–2^ d^–1^ for 28 days, ASTM C1220 test^41,43^), it is still considered highly chemically durable and suitable for the application discussed here.

The current work investigated novel pollucite-based ceramic and glass-ceramic wasteforms as candidates for the immobilization of Cs-loaded IONSIV with high waste loadings. Importantly, the pollucite-based wasteform was designed to allow control of the phase assemblage and Cs-host phase by modifying the synthesis conditions and wasteform chemistry. Further, the wasteform was designed to accommodate variability in the waste chemistry, specifically to produce a common, durable, and flexible phase assemblage irrespective of the amount of Cs present in the IONSIV.

## 2 Experimental Procedure

### 2.1 Materials and Methods

IONSIV R-9120-B ((Na_2–*x*_)(Nb_*x*_Ti_2–*x*_)O_3_SiO_4_·2H_2_O combined with an inert zirconate binder to form the granulated structure) was supplied by Honeywell UOP. The as-received material was contacted with a CsCl solution containing 0.6 M Na_2_SO_4_ (1.8 g/L Cs, 100 mL/g of IONSIV) over 20 h with gentle agitation, targeting a maximum Cs loading capacity of ∼136 mg/g. The unloaded (UL) and Cs-loaded (CsL) IONSIV material were analyzed by X-ray fluorescence (XRF) and inductively coupled plasma mass spectrometry (ICP-MS) to confirm chemical composition.

Although the simple Cs-containing solution used to load the IONSIV material is not necessarily representative of the actual waste solutions to be treated, in the case of the current work, the target waste stream is the actual Cs-loaded IONSIV and not the simple Cs-containing solution. In this regard, IONSIV material has a near-exclusive selectivity for the adsorption of Cs over other ions commonly found in nuclear waste solutions.^58^ For this reason, the Cs-loaded IONSIV prepared here is expected to be a good surrogate for real waste material, and consideration of how other elements impact the waste treatment can be neglected. This approach is similar to that taken for other published work in this field.^23,26,59^ Further, radioactive isotopes of Cs (e.g., Cs-137) will be included in future studies, though the actual isotope of Cs is not expected to influence the phase formation and wasteform design, which is the focus of the current study.

Initial samples were prepared to mimic the IONSIV composition determined for the CsL material using precursor reagents via a modified alkoxide-nitrate route. A full ceramic with a waste loading of 83 wt % denoted as Cs-loaded ceramic (CsL-C), and two Cs-loaded glass-ceramic (CsL-GC) samples with differing glass contents and waste loadings, denoted CsL-GC1 and CsL-GC2, were prepared. Bulk compositions are summarized in Table 1. All chemicals (analytical reagent grade) were purchased from Sigma-Aldrich (Merck) and used as received. Requisite amounts of NaOH, Nb_2_O_5_, Ludox-50 (50%w/w aqueous colloidal suspension of SiO_2_), titanium isopropoxide (TiPT), tetrabutyl zirconate (TBZ), and CsNO_3_ were included to represent the IONSIV material, while Fe(NO_3_)_3_·9H_2_O, calcined Al_2_O_3_, B_2_O_3_, and excess NaOH and SiO_2_ were included as additives to control the formation of the desired phases. The reagents were batched and mixed in a stainless-steel bowl with deionized water. The resulting slurry was stirred and dried overnight on a hot plate at ∼110 °C. The mixture was calcined in an alumina crucible in air at 600 °C for 4 h with a heating/cooling rate of 5 °C/min. The calcined powder was wet ball-milled overnight using yttria-stabilized zirconia grinding media and cyclohexane and subsequently dried at ∼110 °C on a hot plate to produce a fine powder with particle size <10 μm (see Table S1). A portion of the powder was packed into a hardened steel die and cold uniaxially pressed at 180 MPa to produce a green pellet (∼15 mm diameter and 2 mm thickness). The pellet was placed in a platinum crucible seated inside an alumina crucible and then sintered. The sintering profile occurred under an air atmosphere as follows: (a) heat at 5 °C/min from ambient to the designated dwell temperature, (b) hold at the dwell temperature for 6 h, and (c) cool to ambient temperature at 5 °C/min. Four different dwell temperatures were investigated (1100, 1200, 1300, and 1400 °C) for the CsL-C samples. The dwell temperature for sintering CsL-GCs, UL-VAL, and CsL-VAL samples was 1100 °C.

**Table 1:** Bulk Oxide Composition and Sample Identification

Sample Identifier	CsL-C	CsL-GC1	CsL-GC2	UL-VAL	CsL-VAL
Details	Full Ceramic	Glass-Ceramic	Glass-Ceramic	Validation with UL	Validation with CsL
glass content (wt %)	0	3	20	20	20
waste loading (oxide wt %)	83	81	70	a70	a70
**CsL IONSIV Material (wt %)**					
Na_2_O	0.6	0.5	0.5	bUL	bCsL
Nb_2_O_5_	19.2	18.9	16.0		
SiO_2_	13.0	12.8	10.9		
TiO_2_	21.5	21.2	18.0		
ZrO_2_	14.1	13.9	11.8		
Cs_2_O	14.1	13.9	11.8		
**Additives (wt %)**					
Al_2_O_3_	6.1	5.4	6.5	6.5	6.5
Fe_2_O_3_	11.1	11.3	9.6	9.6	9.6
Na_2_O	-	0.4	2.8	2.8	2.8
SiO_2_	-	1.6	10.7	10.7	10.7
B_2_O_3_	-	0.2	1.6	1.6	1.6

a Oxide wt % based on ∼18% loss of water for UL and CsL at 1100 °C.

b Actual amount of IONSIV added needs to consider the inclusion of water (footnote a); so, for a 100 g sample requiring 76.2 g of IONSIV as oxide, 93.0 g of UL or CsL was added.

Validation of the wasteform design was then undertaken using the actual UL and CsL commercial IONSIV material, with the required additives included as oxide or hydroxide salts, and the samples denoted as UL-VAL and CsL-VAL, respectively. All oxide and hydroxide reagents (see Table 1) were ground with UL or CsL IONSIV in a mortar and pestle. These samples were calcined in air at 600 °C for 4 h with a heating/cooling rate of 5 °C/min. The calcined powder was pressed into pellets and sintered in air at 1100 °C for 6 h with a heating and cooling rate of 5 °C/min.

### 2.2 Sample Characterization

The trace elements were quantified by inductively coupled plasma-mass spectrometry (ICP-MS) using a Varian 820-MS mass spectrometry system equipped with nickel cones, a MicroMist ICP nebulizer (Agilent Technologies), a Ryton double-pass Scott-type spray chamber (Agilent Technologies), and an SPS 3 autosampler (Agilent Technologies).

Wavelength dispersive X-ray fluorescence spectrometry was conducted using a Rigaku ZSX Primus IV instrument to analyze the elemental composition using a quantitative empirical calibration method. The sample was prepared for analysis by mixing it with a lithium borate flux and heating it to 1050 °C to create a glass bead. The measured elemental composition for CsL-C, CsL-GC1, and CsL-GC2 was determined by XRF, and the results are in reasonable agreement with the targeted compositions, noting the somewhat higher Al_2_O_3_ content of CsL-C (see Table S2).

The mineralogy was determined by X-ray diffraction (XRD). X-ray powder diffraction patterns were obtained using a Philips PW1050 diffractometer (PANalytical Ltd., Almelo, The Netherlands) with CuKα radiation, an angular range of 5°–80° 2θ, a step size of 0.03° 2θ, and a step time of 5 s. Phase abundances were determined by Rietveld refinement analysis using HighScore Plus software.^60^

The bulk density and apparent porosity of the samples were determined according to ISO 18754–2020 by liquid displacement (Archimedes’ method^61^). The analysis was performed in duplicate, and the results reported are the average of the measurements. The true density was determined using an Anton Paar Ultrapyc 5000 gas pycnometer. Helium gas was used for cell pressurization. Measurement uncertainties are estimated from the standard deviation of the mean of replicate results at the 95% confidence level.

Thermogravimetric analysis (TGA) and differential thermal analysis (DTA) were conducted simultaneously from room temperature up to 1300 °C. ∼100 mg of powder or monolith was investigated by TG-DTA with a heating rate of 10 °C/min by using a NETZSCH model STA 449 F3 Jupiter apparatus equipped with a SiC furnace. Alumina crucibles were used along with alumina powder as the reference.

The morphology and elemental composition of samples were examined by using scanning electron microscopy (SEM) and transmission electron microscopy (TEM) with energy-dispersive X-ray spectroscopy (EDS). Samples were examined using a Zeiss Ultra Plus (SEM-EDS) (Carl Zeiss NTS GmbH, Oberkochen, Germany), operating at 15 kV and equipped with an X-Max 170 mm^2^ SDD X-ray microanalysis system (Oxford Instruments, Abingdon, Oxfordshire, UK). The samples were cold-mounted in epoxy resin, and final polishing was performed utilizing diamond-impregnated polishing pads (3 and 1 μm). The polished samples were coated with ∼10 nm of carbon using a carbon evaporative coater. TEM samples were prepared by grinding fine particle fragments under ethanol with a mortar and pestle. The solution containing the fragments was drawn up via pipet and deposited onto holey carbon film supported on TEM copper mesh grids. Microscopy was carried out using a JEOL 2200 FS TEM operated at 200 kV, with images and diffraction patterns collected by Gatan Orius and Ultrascan cameras. The TEM was fit with an Oxford X-Max energy-dispersive X-ray detector (EDS), 80 mm^2^, with data analysis performed via Oxford INCA, version 4.15.

Chemical durability testing was conducted according to the standard ASTM C1285 Product Consistency Test (PCT), Test Method B,^62^ using the following experimental conditions: ASTM Type I deionized water leachant, a 7-day test period, PFA TFE-fluorocarbon test vessels, a 0.150–0.075 mm particle size range, a 90 °C test temperature, and a 10 cm^3^ g^–1^ leachant volume/sample mass ratio. Leachate solutions were analyzed for elemental composition by ASTM C1109 – analysis of aqueous leachates from nuclear waste materials using inductively coupled plasma atomic emission spectroscopy.

## 3 Results and Discussion

### 3.1 IONSIV Composition

The elemental composition of the UL and CsL IONSIV was first determined by XRF analysis and separately (for UL IONSIV) via digestion in oxalic acid and ICP-MS (see Table S3). The elemental quantities were used to derive an empirical formula of (Na_0.4_)(Nb_0.6_Ti_1.4_)Si_1.07_O_7_·*x*H_2_O, which is consistent with previously reported results,^8,9^ noting that water content was not measured. Compositional analysis also revealed the presence of Zr, which arises from the Zr(OH)_4_ organic binder used to coagulate the IONSIV powder into beads. Importantly, there was no evidence from XRF data of significant Cs release from CsL IONSIV following the various thermal treatments (no heat treatment, 600, 800, and 1100 °C) up to 1100 °C. The Cs composition of the CsL IONSIV was determined to be ∼17 wt % (oxide wt %) from XRF analysis, replacing sodium (∼4.5 wt % Na_2_O in UL IONSIV and 0.5 wt % in CsL IONSIV). The XRF results for the UL and CsL IONSIV (after thermal treatment at 800 °C) were used to guide the formulation of the initial candidate wasteform samples, prior to validation of the wasteform design with the actual IONSIV materials.

### 3.2 Thermal Treatment of IONSIV

TGA-DTA was performed under an argon atmosphere for the UL and CsL IONSIV (see Figure S1). To assist in the comprehension of the UL and CsL IONSIV thermal profiles, SEM-EDS (Figures S2 and S3) and XRD (Figure S4) analyses were also carried out by following various thermal treatments (before sintering and after sintering at 600 °C, 800 °C, and 1100 °C for 1 h in air). In both samples, the major mass loss at temperatures below 600 °C was attributed to the desorption of bound water,^8,9,17^ with corresponding endothermic peaks in the DTA at 270 and 240 °C for the UL (∼18.9 wt % mass loss) and CsL (∼13.7 wt % mass loss) IONSIV, respectively. A second mass loss in the UL sample between 300 °C and 600 °C was attributed to the conversion of the Zr(OH)_4_ binder to ZrO_2_.^17^ Water loss at temperatures of <500 °C in both samples was associated with amorphization. Exothermic peaks between 700 and 1250 °C in UL and CsL IONSIV were due to the high-temperature crystallization of various phases, including ZrTiO_4_, SiO_2_, and TiO_2_ in the UL sample,^63^ and CsTi_2_NbO_7_ and Cs_2_Nb_6_TiO_18_ phases in the CsL sample. An endothermic peak at 1200 °C, which corresponds to a 0.2 wt % weight loss in the CsL sample, may be due to the sublimation of Cs_2_O in the form of gas.^64^

### 3.3 Wasteform Design

#### 3.3.1 Full Ceramic Wasteform Design, CsL-C

The full ceramic wasteform design (CsL-C) targeted pollucite (CsAlSi_2_O_6_) for Cs incorporation. Al_2_O_3_ was included as an additive (Cs:Al molar ratio = 1.0:1.2) to promote the formation of pollucite. Excess Al was expected to form Al_2_O_3_ as a phase compatible with pollucite. Fortuitously, pollucite also provides a suitable phase for the incorporation of minor amounts of Na in the waste,^65^ as well as the majority of the Si present (∼92% of the Si inventory in the case of stoichiometric CsAlSi_2_O_6_). Rutile and zirconia were both considered stable and compatible phases in the design with pollucite, and an equimolar amount of Fe^3+^ to Nb^5+^ was included to promote the formation of a solid solution with Ti as (Ti_1–2*x*_Nb^5+^_*x*_Fe^3+^_*x*_)O_2_, where Nb is substituted for Ti and Fe provides charge balancing. The wasteform design maximizes the waste loading (83 wt % on an oxide basis) with the only additives being Al_2_O_3_ and Fe_2_O_3_. The samples were sintered at various temperatures up to 1400 °C in air for 6 h and were characterized using XRD and SEM-EDS.

**Figure 1:**
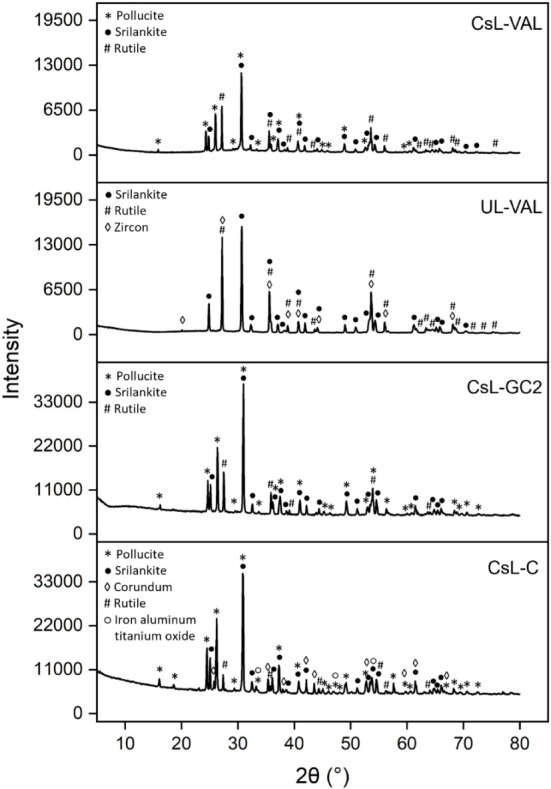
XRD patterns of CsL-C sintered at 1400 °C, CsL-GC2, UL-VAL IONSIV, and CsL-VAL IONSIV sintered at 1100 °C in air for 6 h.

**Figure 2:**
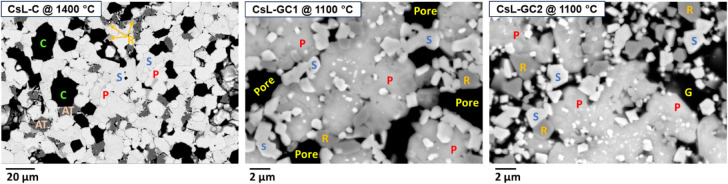
SEM images of CsL-C and CsL-GCs samples. The phases are labeled as (P) pollucite, (S) srilankite, (R) rutile, (C) corundum, (AT) aluminum titanate, and (G) glass. Pores are labeled in CsL-GC1.

XRD analysis (Figure 1) showed the formation of a multiphase ceramic system for CsL-C following consolidation at 1400 °C. A similar phase formation was observed irrespective of the consolidation temperature between 1100 and 1400 °C (see Figure S5). The main phases identified in the XRD pattern were pollucite ((Cs, Na)AlSi_2_O_6_, tetragonal, *I*41/*acd*, JCPDS 01–077–1124), srilankite (ZrTi_2_O_6_, orthorhombic, *Pbcn*, JCPDS 01–075–1739), and minor amounts of corundum (Al_2_O_3_, rhombohedral, *R*3̅*c*, JCPDS 01–075–0782), rutile (TiO_2_, tetragonal, *P*42/*mnm*, JCPDS 01–073–1765), and trace amounts of iron aluminum titanate (orthorhombic, *Cmcm*, *P*42/*mnm*, JCPDS 01–076–1157). The XRD data were in good agreement with the phases identified by SEM-EDS (Figure 2 and Table 2). The consolidation temperature did not impact the elemental composition of each phase as determined by SEM-EDS analyses, and average phase compositions for all ceramic samples are summarized in Table 2.

**Table 2:** Summary of the Average Composition of Phases from the SEM-EDS Analysis for CsL-C, CsL-GCs, UL-VAL, and CsL-VAL Wasteforms

Phase	CsL-C	CsL-GC1 and CsL-GC2	UL-VAL	CsL-VAL
	Composition from SEM-EDS			
apollucite	Cs_0.95_AlSi_2_O_6_	Cs_0.85_Al_0.87_Si_2_O_6_	NP	Cs_0.93_AlSi_2_O_6_
bsrilankite	(Ti_0.37_Zr_0.20_Nb_0.25_Fe_0.15_)O_2_	(Ti_0.39_Zr_0.22_Nb_0.19_Fe_0.18_)O_2_	(Ti_0.45_Zr_0.29_Nb_0.14_Fe_0.12_)O_2_	(Ti_0.40_Zr_0.24_Nb_0.17_Fe_0.16_)O_2_
crutile	(Ti_0.45_Nb_0.25_Fe_0.20_)O_2_	(Ti_0.55_Nb_0.18_Fe_0.17_)O_2_	(Ti_0.62_Nb_0.15_Fe_0.14_)O_2_	(Ti_0.57_Nb_0.18_Fe_0.18_)O_2_
dglass(Cs content in at.%)	NP	Na_0.70_Al_0.45_B_0.50_Si_2_O_6_(0.85 at.%)	Na_0.62_Al_0.30_B_0.50_Si_2_O_6_	Na_1.20_Al_0.60_B_0.50_Si_2_O_6_(0.75 at.%)
additional phase	Al_2_O_3_	NP	eZrSiO_4_, Al_0.60_Fe_2_Ti_0.90_O_5_, Al_2_O_3_	NP

a Pollucite contains minor Na (<0.06 f.u.), Ti (<0.13 f.u.), and Fe (<0.15 f.u.) in all samples.

b For CsL-C, srilankite contains minor Al (<0.05 f.u.).

c Rutile contains minor Al (<0.05 f.u.) and Zr (<0.10 f.u.) in all samples.

d For CsL-GC, UL-VAL, and CsL-VAL, glass contains trace Ti (<0.15 f.u.), Fe (<0.20 f.u.), Nb (<0.12 f.u.). CsL-GC2 and CsL-VAL contain Cs (∼0.07 f.u.). For CsL-GC1, glass contains trace Ti (∼0.45 f.u.), Fe (∼0.38 f.u.), Zr (∼0.18 f.u.), Nb (∼0.26 f.u.), and Cs (∼0.11 f.u.).

e Zircon contains minor Ti (<0.15 f.u.) and Nb (<0.05 f.u.).

Figure 2 provides phase identification and microstructure of CsL-C sintered at 1400 °C. The sample sintered at 1100 °C displayed significantly higher porosity compared to the samples sintered at relatively higher temperatures. At the lower sintering temperature (1100 °C), pollucite (CsAlSi_2_O_6_) was characterized as the main, mid-gray phase. Small, submicron-sized, similar-contrast particles of srilankite were associated with the pollucite. The density increased with a temperature of above 1100 °C (see Figure S6). Also, at higher sintering temperatures, islands of dark-contrast corundum (Al_2_O_3_) with diameters of 10–20 μm were observed. XRD and SEM results confirm that the targeted phase assemblage was achieved. The addition of Al_2_O_3_ facilitated the formation of pollucite to incorporate the Cs and Si components of the waste, and the addition of Fe_2_O_3_ provided charge balancing, allowing the incorporation of Nb^5+^ within the srilankite and rutile phases.

The bulk density measurements for the CsL-C samples (Table 3) confirm observations from the SEM micrographs. CsL-C sintered at 1100 °C is extremely porous, with relatively low bulk density and high apparent porosity. Bulk density increased with sintering temperature (1200–1400 °C), though a subtle decrease in bulk density was observed at 1400 °C relative to the 1300 °C sample. Apparent porosity was determined to be 1.10% for CsL-C sintered at 1300 °C, which was the lowest value determined for all CsL-C samples studied.

**Table 3:** Density and Porosity Values for the CsL-C and CsL-GC Wasteforms

Sample	CsL-C				CsL-GC1	CsL-GC2
sintering temperature (°C)	1100	1200	1300	1400	1100	1100
atrue density (g/cm^3^)	-	-	4.174 ± 0.005	-	4.89 ± 0.13	3.92 ± 0.05
bbulk density (g/cm^3^)	2.14 ± 0.05	3.56 ± 0.05	3.85 ± 0.05	3.73 ± 0.05	2.58 ± 0.06	3.40 ± 0.06
bapparent porosity (%)	47.6 ± 4.00	9.90 ± 0.50	1.10 ± 0.20	4.90 ± 0.50	36.5 ± 1.70	3.40 ± 1.80

a The reported estimate of measurement uncertainty was calculated at an approximately 95% confidence level.

b Reported numbers are the average of duplicate samples.

#### 3.3.2 Glass-Ceramic Wasteform Design, CsL-GC

Similarly to the full ceramic, glass-ceramics (GCs) targeted pollucite for the immobilization of Cs. Again, an equimolar amount of Fe^3+^ to Nb^5+^ was included to promote srilankite and rutile, in combination with the Ti and Zr present in the waste. Sodium aluminoborosilicate glass (NaAl_0.5_B_0.5_Si_2_O_6_) was targeted at either 3 or 20 wt % to investigate the impact of glass content on properties. The glass composition was based on previous work formulating fluorite, pyrochlore, and zirconolite GCs.^28,66^ Glass not only provides flexibility to the wasteform design, as it can incorporate small amounts of all the waste elements, but also can simplify the processing requirements. The addition of glass, however, reduces waste loading slightly relative to CsL-C, with waste loadings of 81 wt % (CsL-GC1, 3 wt % glass) and 70 wt % (CsL-GC2, 20 wt % glass) achieved. GC samples were sintered at 1100 °C to allow the assessment of densification from glass inclusion relative to CsL-C.

XRD analysis (Figure 1) showed the formation of pollucite, srilankite, and rutile as the main phases in both GCs, which matched the reference patterns for CsL-C. Glass content did not have a significant effect on crystalline phase formation, though it significantly inhibited the formation of corundum and aluminum titanate phases relative to CsL-C. Microstructural and EDS analysis (Figure 2 and Table 2) confirmed the XRD results for both GCs. Glass content did not impact the elemental composition of each of the phases formed, and the resulting average phase compositions for each GC are provided.

From the SEM micrographs (Figure 2), it appeared that the inclusion of glass reduced the porosity under similar sintering conditions (1100 °C for 6 h). This was confirmed via density measurements (Table 3), which returned apparent porosity results of 47.6, 36.5, and 3.40% for samples with 0, 3, and 20 wt % glass added. In addition, the Cs content of the glass phase was extremely low (the detection limit of Cs in glass in SEM is 0.1 wt %^67^), with the vast majority incorporated into the pollucite crystalline phase, as targeted. SEM-EDS analysis confirmed that 99% and 93% of the Cs inventory was partitioned to the pollucite phase for CsL-GC1 and CsL-GC2, respectively (see Table S4 and Note 1 in the Supporting Information). From a wasteform design perspective, partitioning of Cs into pollucite rather than glass is desirable, given its greater chemical durability.

#### 3.3.3 Wasteform Chemical Durability

The chemical durability of the ceramic and glass-ceramics was assessed using the ASTM C1285 test method,^62^ and the results are reported in Table 4 together with ASTM C1285 results previously published in the literature for other candidate glasses and ceramics, where available. Elemental release from the GCs is incongruent, with significantly lower normalized losses found for the major ceramic components (Cs, Ti, Nb, Fe, and Zr) relative to the glass-forming elements Na, B, Si, and Al. Further, Cs release from the GCs was relatively low (98 ± 12 and 39.1 ± 4.6 mg/m^2^ for CsL-GC1 and GC2, respectively), indicating the effective immobilization of Cs within the more durable pollucite phase. The sample with higher glass content (CsL-GC2) displayed lower Na and B normalized mass loss relative to CsL-GC1, which may be due to its higher density. The release of Na from the two GCs is comparable to that found for the reference glass (“Glass”,^68^ 30 wt % CST waste loading), noting that the GCs have significantly enhanced waste loading and improved Cs retention. Cs normalized mass loss from CsL-GC2 showed an almost 7-fold improvement when compared to the reference HIPed ceramic previously reported.^26^ This was found similarly to the full ceramic sample, CsL-C, with regard to Cs; however, a relatively high normalized mass loss of Na (13800 ± 2800 mg/m^2^) for CsL-C was due to the absence of a suitable host phase for Na, justifying the inclusion of glass in the GC design.

**Table 4:** ASTM C1285 Chemical Durability Test Results for CsL-C, CsL-GC1, and CsL-GC2 (1100 °C in Air for 6 h) and Reference Ceramic and Glass^a^

	Normalized Mass Loss (NL_i_), mg/m^2^ after 7 days				
Element	CsL-GC1	CsL-GC2	CsL-C	Reference Ceramic^26^	bReference Glass^68^
**Al**	460 ± 60	127.2 ± 3.7	516 ± 30	N/A	N/A
**B**	3080 ± 140	184 ± 7	N/A	N/A	856
**Cs**	98 ± 12	39.1 ± 4.6	180 ± 60	265 ± 10.5	N/A
**Fe**	8.4 ± 1.1	0.96 ± 0.33	0.63 ± 0.16	N/A	N/A
**Na**	13 800 ± 2900	1200 ± 120	13 800 ± 2800	777 ± 30	535
**Nb**	13 ± 25	1.14 ± 0.10	0.296 ± 0.029	0.7 ± 0.7	NR
**Si**	950 ± 140	142 ± 8	970 ± 60	286 ± 3.5	NR
**Ti**	7.1 ± 1.9	0.44 ± 0.08	1.16 ± 0.23	0.7 ± 0.7	NR
**Zr**	8 ± 9	2.50 ± 0.22	0.026 ± 0.005	1.4 ± 0.7	NR

a NR = not reported; N/A = not available in the wasteform.

b Normalized elemental mass loss values for 30% CST waste loading glass were calculated based on the reported normalized concentration and density results in ref (68) using the formulas provided in ASTM C1285 sections 25.4–25.5, assuming that the standard particle size and leachant volume values were employed as per ASTM C1285, Test Method A.

It is also useful to compare Cs release rates in these candidate wasteforms for IONSIV immobilization with pure Cs-containing phases, noting that fabrication conditions, elemental compositions, and leach testing methodologies may be different. The Cs release rates for the samples prepared in this study (0.04–0.2 g/m^2^) are very low and comparable to those reported for hollandite (∼0.2 g/m^2^),^69^ Synroc C (∼0.7 g/m^2^),^70^ sodium zirconium phosphate (0.01–0.3 g/m^2^),^49^ and pollucite (0.2–2 g/m^2^)^71^ in 7-day leach tests.

#### 3.3.4 Wasteform Validation

Given the relatively high waste loading, acceptable phase formation, relatively low sintering temperature required, and the promising aqueous durability results, the GC with 70 wt % waste loading and 20 wt % glass (CsL-GC2) was selected to progress to a wasteform validation study. In this study, phase formation was assessed using previously prepared UL and CsL IONSIV materials (nonradioactive Cs) by simply mixing 70 wt % of the IONSIV (either UL or CsL) with the required additives (Al_2_O_3_, Fe_2_O_3_, B_2_O_3_, NaOH, and SiO_2_) according to the composition provided in Table 1, with sintering at 1100 °C. Given the limited powder processing steps during synthesis, the wasteform design was assessed for its ability to promote the required phase formation without precursor powder milling steps, and to accommodate waste variations. To establish if a common phase assemblage will form irrespective of the amount of Cs loaded onto the IONSIV adsorbent, extremes of Cs loading (saturated and unloaded) were examined. The samples were identified through the validation study as UL-VAL for the unloaded IONSIV GC sample and CsL-VAL for the Cs-loaded IONSIV GC sample.

Figure 1 provides the XRD patterns for UL-VAL and CsL-VAL, and both samples showed the formation of srilankite and rutile as predominant crystalline phases. The UL-VAL sample showed trace zircon (ZrSiO_4_, tetragonal, 141/*amd*, JCPDS 01–083–1379). Zircon is a naturally occurring mineral that is extremely durable and has long been established for nuclear waste immobilization,^72^ and its trace inclusion is acceptable. The CsL-VAL sample additionally showed the formation of pollucite for Cs immobilization. A quantitative phase analysis for CsL-GC1, CsL-GC2, UL-VAL, and CsL-VAL showed srilankite to be the most abundant phase in almost all samples (41–48%) with pollucite being the second most abundant phase in the Cs-loaded samples (29–36%). CsL-VAL showed results similar to CsL-GC samples, though with a slightly higher rutile content (30% compared to 16–20%). Only minor amounts of zircon (1.3%) were present in the UL-VAL sample, which also showed a high rutile abundance of 51.7% (see Table S4).

**Figure 3:**
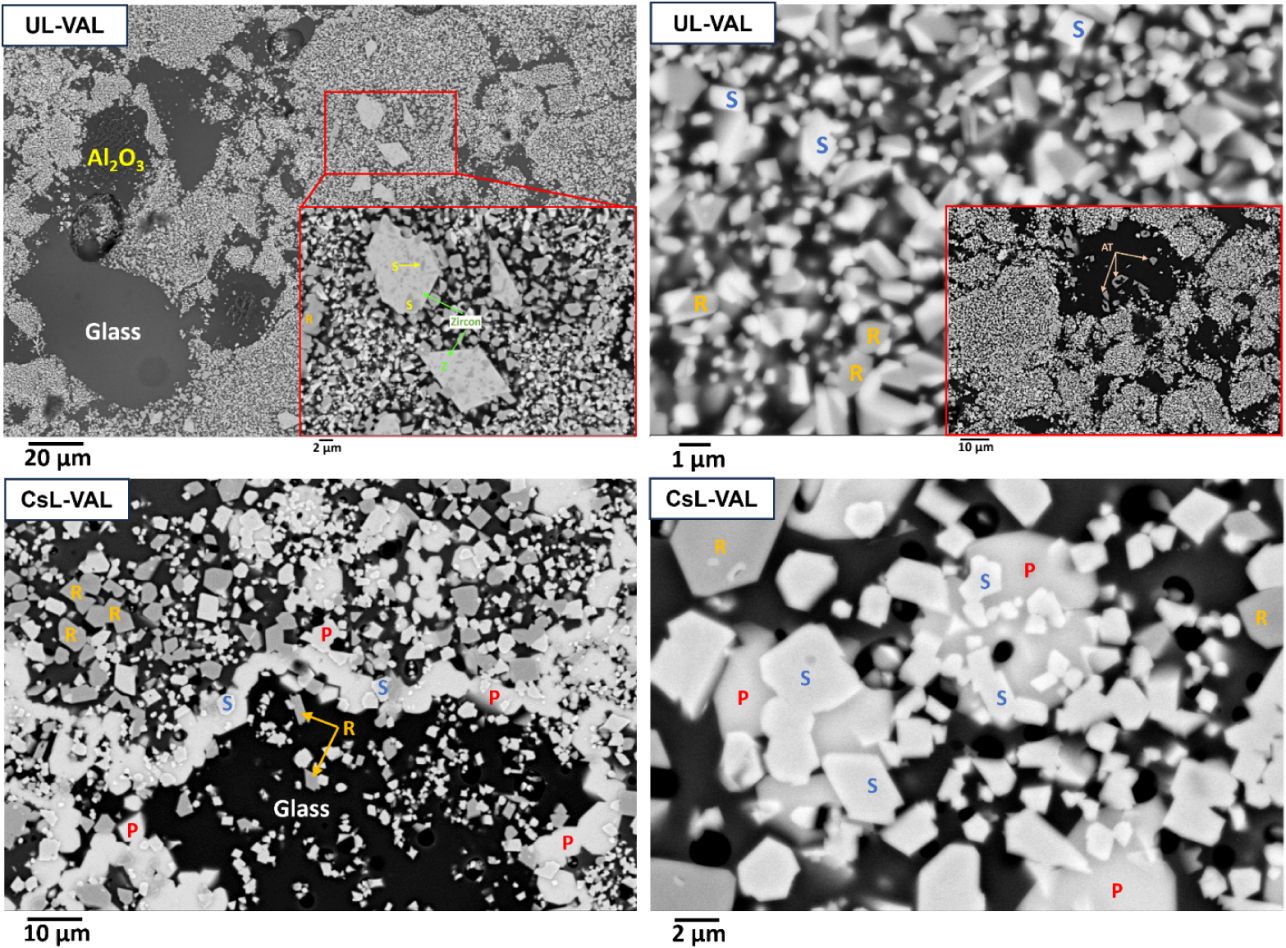
SEM images of UL-VAL and CsL-VAL samples sintered at 1100 °C. The phases are labeled as (P) pollucite, (S) srilankite, (R) rutile, (Z) zircon, (AT) aluminum titanate, and glass.

SEM images (Figure 3), EDS analysis (Table 2), and EDS spectra (see Figures S7 and S8) for UL-VAL and CsL-VAL are consistent with XRD data. The UL-VAL sample showed the formation of srilankite, rutile, zircon, and trace aluminum titanate in the SEM image. Glass was also identified with a composition varying in the range of Na_0.6–0.9_Al_0.3–0.7_B_0.5_Si_2_O_6_ (noting that B is unmeasurable by SEM-EDS and therefore inferred). The glass also contained minor amounts of titanium, iron, and Nb. Minor Al_2_O_3_ was crystallized in glass-rich areas of UL-VAL as a result of the excess Al_2_O_3_ included in the design to ensure pollucite formation when Cs was present. CsL-VAL displayed an additional pollucite phase and a glass composition in the range of Na_1.2–1.5_Al_0.6–1.5_B_0.5_Si_2_O_6_. As with the UL-VAL sample, the glass contained minor amounts of Ti, Fe, and Nb, and Cs. Targeted phase compositions were measured irrespective of the Cs loading on the IONSIV starting material.

**Figure 4:**
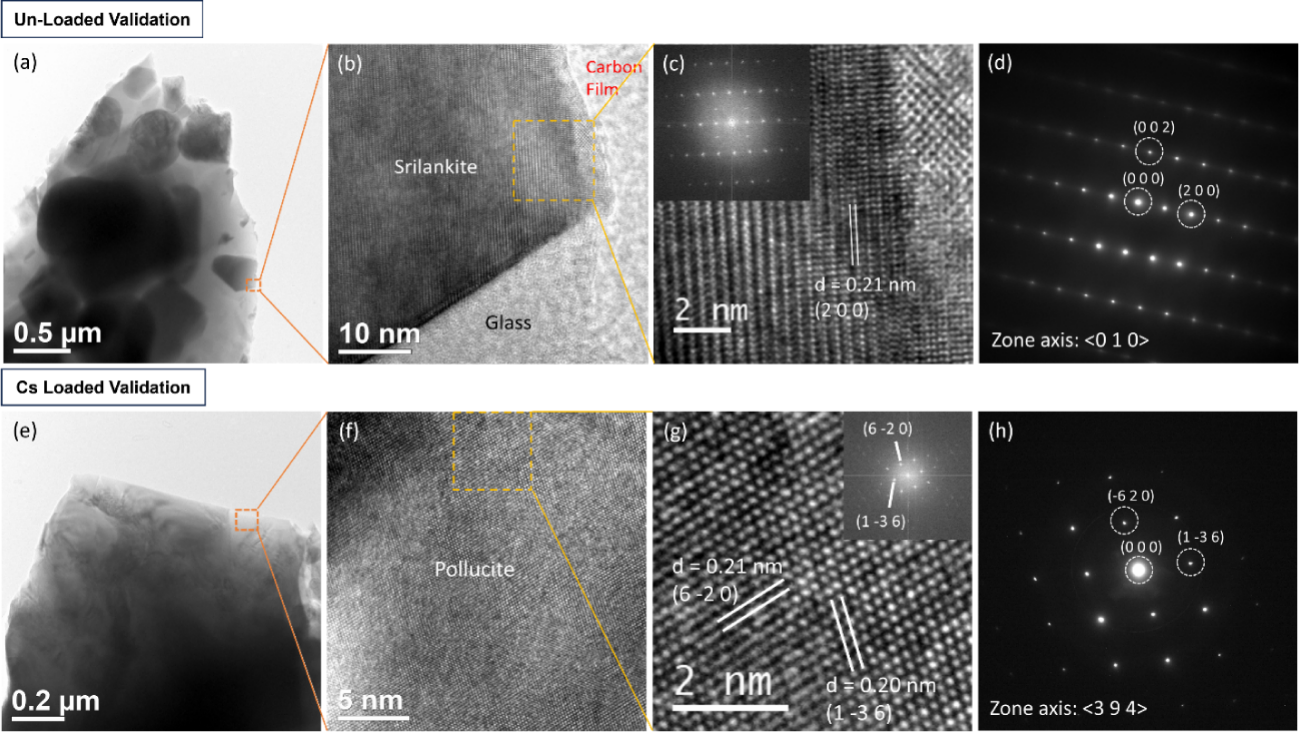
(a, e) TEM bright-field image, (b, f) high-resolution (HRTEM) image showing the layer spacing, (c, g) lattice resolved HRTEM image with the inset showing 2D fast Fourier transform (FFT), and (d, h) SAED pattern of the crystal fragment of orthorhombic srilankite and tetragonal pollucite phases for UL-VAL and CsL-VAL samples. The SAED pattern of srilankite in UL-VAL and pollucite in CsL-VAL was viewed down the <0 1 0> zone axis and <3 9 4> zone axis, respectively.

TEM-EDS analysis was undertaken on UL-VAL and CsL-VAL to confirm the phase allocation and SEM-EDS results (Figure 4). TEM-EDS analysis for UL-VAL and CsL-VAL (Table S5) provided compositions that were in good agreement with SEM-EDS data. The TEM data for UL-VAL confirmed the formation of srilankite with a *d*-spacing of 0.21 nm for the crystal lattice plane (2 0 0). This was marginally lower (∼10%) than the ideal value according to the XRD reference pattern (ZrTi_2_O_6_, 0.23 nm, JCPDS 01–075–1739). The XRD peaks in Figure 1 showed a similar shift from the ideal reference pattern of srilankite and returned a *d*-spacing of 0.23 nm for the (2 0 0) plane (XRD diffraction peak at 38° 2θ). This may be a result of the incorporation of Nb^5+^ and Fe^3+^ into the lattice of srilankite, as these elements have different ionic radii compared with Ti^4+^ and Zr^4+^.^73^ The selected area electron diffraction (SAED) pattern of this phase, as viewed down the <0 1 0> zone axis with the Bragg maxima for the (0 0 2) and (2 0 0) crystal lattice planes highlighted, also confirmed its identity as srilankite (orthorhombic crystal structure with a space group of *Pbcn*). TEM data for CsL-VAL (Figure 4) confirmed the formation of pollucite with crystal lattice planes (1 3̅ 6) and (6̅ 2 0) and corresponding *d*-spacings of 0.20 and 0.21 nm, respectively. These align with the (6 3 1) (*d*-spacing of 0.20 nm) and (6 2 0) (*d*-spacing of 0.21 nm) diffraction peaks of the ideal XRD reference pattern ((Cs, Na)AlSi_2_O_6_, JCPDS 01–077–1124). The SAED pattern of this phase, as viewed down the <3 9 4> zone axis with Bragg maxima for the (1 3̅ 6) and (6̅ 2 0) crystal lattice planes highlighted, also confirmed its identity as pollucite (tetragonal crystal structure with a space group of *I*41/*acd*). TEM-EDS analysis confirmed that ∼93% (Table S4) of the Cs inventory in CsL-VAL was partitioned to the pollucite phase, in agreement with that found for CsL-GC2.

Consideration of the targeted Cs-137 loading onto the IONSIV is required, as it generates high radiation fields and, consequently, appreciable amounts of radiogenic heat, which must be considered in wasteform design, waste classification, and finally, disposal. For example, a theoretical wasteform package of 5 L (e.g., a 5 L HIPed canister of CsL-GC2 containing 19.5 kg of the material) with 0.002% w/w Cs-137 loading on the IONSIV would have an activity concentration of 176 GBq/L, which is typical of US class-C waste (4600 Ci/m^3^, or 170 GBq/L) (see Note 2 in the Supporting Information). Such a theoretical waste package would have a heat output of ∼0.11 W, an activity concentration of ∼4.5 × 10^7^ Bq/g, and a gamma dose rate on contact of 3 Gy/h (estimated using Microshield Pro 13.10X software). At these levels of activity and heat generation, the package may be defined as ILW according to International Atomic Energy Agency (IAEA) guidelines.^74^ Higher Cs-137 loadings on the IONSIV may result in such wasteforms being classified as HLW, thus requiring disposal at greater depths.^74^ For example, if the Cs loading rate was 0.1% w/w, a 5 L wasteform package would generate approximately 6 W of radiogenic heat, with a predicted activity concentration of 2.3 × 10^9^ Bq/g and a modeled dose rate on contact of ∼150 Gy/h. Irrespective of the loading, the current wasteform design was demonstrated for such ranges of Cs-137 waste loadings. The selected candidate wasteform maintained a very high waste loading of 70 wt % and the inclusion of glass provided a chemically flexible and simpler-to-process material. Importantly, the tailored design ensured the production of a consistent phase assemblage, irrespective of Cs-loading on the IONSIV, with Cs partitioned (>90% of the inventory) into the more chemically durable pollucite phase. This was successfully demonstrated in the validation study, which considered the extremes of Cs loading, i.e., Cs-saturated IONSIV and unloaded material (CsL-VAL and UL-VAL, respectively). The results of chemical durability testing indicated an improved design relative to alternative candidate wasteforms for the immobilization of Cs-loaded IONSIV. The samples also showed high mechanical durability, with compressive strength values between 50 and 300 MPa^75^ (see Figure S9). Table 5 provides a detailed comparison of this wasteform and treatment approach to the state of the art, noting that no treatment option is currently in place.

**Table 5:** Wasteform and Processing Options for the Treatment of Radioactively Contaminated CST

Wasteform	Matrix	Processing Technique	Waste Loading (wt % CST)	C onsiderations
**extended storage**(17)	Cs-exchanged IONSIV.No encapsulation or immobilization.	none	no loading	Need substantial further work to assess: robustness of the column package/required shielding/thermal management strategies/ventilation requirements/gas generation rates/impact of radiation damage/unknown chemical durability/accessibility for future treatment.
**resin**(76)	Phenol formaldehyde binding agent.Encapsulation.	column infiltration (180 °C)	Cs-CST: resin mass ratio = ∼3:2	Resin added to column in situ to bind beads.
				Compressive strength equal to or exceeding cement.
				Durable wasteform (more work required to allow comparison to other technologies).
				Further work required to understand long-term stability and to allow comparison.
**cement**(23)	Ordinary portland cement (OPC) + blast furnace slag(BFS)/fly ash. Encapsulation.	manual mixing	20	Mature and internationally accepted technology successfully operated at industrial scale at many sites.
				Cementitious wasteforms are normally considered for LLW and some ILW.
				Substantially less durable relative to glass, ceramic and glass-ceramic wasteforms.
				Needs to consider hydrogen generation from radiolysis.
				Low waste loadings relative to glass, ceramic and glass-ceramic wasteforms.
				Low temperature process resulting in less off-gas requirements and secondary wastes.
**glass**(33,34,77)	Borosilicate glass.Immobilization.	vitrification(1150 °C)	33	Mature Technology. Industrially operated for HLW on several sites, e.g., US, Germany, Belgium, Russia, and Japan.
				Off-gas treatment system designed to capture volatilized radionuclides (e.g., Cs-137) producing secondary wastes.
				Tolerance to crystalline phases requires evaluation and may limit waste loading.
				Moderate waste loading. Higher waste loadings (perhaps up to 50 wt %) may be possible but require further investigation.
				Durable wasteform.
**ceramic**(26)	Multiphase ceramic.Immobilization.	HIP(1100 °C and 190 MPa)	100	HIP developed in Australia since 1995. Industrial facility for ILW currently undergoing commissioning.
				High waste loading.
				No control over phase assemblage.
				Uncontrolled formation of poorly durable Cs-bearing phases.
**glass-ceramic**(this work)	Borosilicate glass + rutile + srilankite + pollucite.Immobilization.	HIP(1150 °C and 100 MPa)	70	HIP developed in Australia since 1995. Industrial facility for ILW currently undergoing commissioning.
				High waste loading.
				Controlled phase assemblage irrespective of Cs-loading on the IONSIV.
				High chemical durability (improved relative to glass and ceramic options).
				Removes potential for radionuclide volatilization and corrosive chemical emissions into the off-gas system during high-temperature consolidation.
				Minimizes secondary wastes.
				Removes melter corrosion problems as processing unit is separated from the waste.

There are a range of positive environmental implications that would be realized by implementing this proposed novel wasteform solution. First, the tailored and novel glass-ceramic wasteform has high chemical durability compared with current alternative options. Given that the initial rate-limiting factor for radionuclide release to the environment is dependent on the chemical durability of the wasteform within the disposal site, the best way to reduce environmental risk and radionuclide release to the biosphere is to optimize the wasteform by careful design for the waste in question. This has been successfully achieved in the current work. Second, by increasing the waste loading, the volume of the wasteform is reduced, resulting in both reduced life-cycle costs, transport, and geological repository volume requirements. Increasing the waste loading, however, typically results in a less durable wasteform and, therefore, a higher environmental risk. The current work balances waste loading and chemical durability for an optimized solution. Third, the wasteform is compatible with HIP processing, and an important advantage of this technology is that the theoretical density of the material can be achieved with minimum temperature, thereby adding to the overall strength and chemical durability of the wasteform. Further, environmental impacts from radionuclide volatilization during high-temperature processing and subsequent secondary waste production are eliminated as consolidation occurs within sealed metal HIP canisters.

## Supporting Information Available

The particle size analysis results for the calcined and milled powders (Table S1); the target and measured elemental wt % for CsL-C and CsL-GCs samples (Table S2); the experimentally determined bulk compositions for unloaded and Cs-loaded IONSIV (Table S3); data to support Cs-partitioning results and phase abundance (Table S4); elemental analysis results for UL-VAL, and CsL-VAL wasteforms (Table S5); thermal gravimetric analysis and differential thermal analysis curves for Cs-loaded and unloaded IONSIV samples (Figure S1); scanning electron micrographs of unloaded IONSIV under thermal treatment at a range of temperatures (Figure S2); scanning electron micrographs of Cs-loaded IONSIV under thermal treatment at range of temperatures (Figure S3); X-ray diffraction patterns for Cs-loaded and unloaded IONSIV under thermal treatment at a range of temperatures (Figure S4); X-ray diffraction patterns for CsL-C sintered at various temperatures (Figure S5); scanning electron micrographs for the CsL-C sintered at various temperatures (Figure S6); energy-dispersive X-ray spectroscopy data for UL-VAL (Figure S7); energy-dispersive X-ray spectroscopy data for CsL-VAL (Figure S8); compressive strength results for CsL-C, CsL-GCs, and UL-VAL samples (Figure S9); the impact of glass content on Cs-partitioning calculations (Note 1); a list of assumptions used for the activity concentration, heat output, and contact gamma dose calculations (Note 2) (PDF)

## References

[ref1] International Atomic Energy Agency Application of Ion Exchange Processes for the Treatment of Radioactive Waste and Management of Spent Ion Exchangers; International Atomic Energy Agency, 2002. AC03480727, A

[ref2] Figueiredo B. R.; Cardoso S. P.; Portugal I.; Rocha J.; Silva C. M. Inorganic Ion Exchangers for Cesium Removal from Radioactive Wastewater. Sep. Purif. Rev. 2018, 47 (4), 306–336. 10.1080/15422119.2017.1392974.

[ref3] Luca V.; Hanna J. V.; Smith M. E.; James M.; Mitchell D. R.; Bartlett J. R. Nb-Substitution and Cs^+^ Ion Exchange in the Titanosilicate Sitinakite. Microporous Mesoporous Mater. 2002, 55 (1), 1–13. 10.1016/S1387-1811(02)00353-0.

[ref4] Tripathi A.; Medvedev D. G.; Nyman M.; Clearfield A. Selectivity for Cs and Sr in Nb-substituted Titanosilicate with Sitinakite Topology. J. Solid State Chem. 2003, 175 (1), 72–83. 10.1016/S0022-4596(03)00145-2.

[ref5] Chitra S.; Sudha R.; Kalavathi S.; Mani A.; Rao S.; Sinha P. Optimization of Nb-Substitution and Cs^+^/Sr^+2^ Ion Exchange in Crystalline Silicotitanates (CST). J. Radioanal. Nucl. Chem. 2013, 295, 607–613. 10.1007/s10967-012-1812-0.

[ref6] Al-Attar L.; Dyer A.; Paajanen A.; Harjula R. Purification of Nuclear Wastes by Novel Inorganic Ion Exchangers. J. Mater. Chem. 2003, 13 (12), 2969–2974. 10.1039/b308060a.

[ref7] Fu C.; Wei X.; Lian J.; Cheng J.; Zhu S.; Yan M. A Facile Synthesis of Polyacrylic Acid–Ammonium Phosphomolybdate Microspheres for The Highly Selective Removal of Cesium. J. Radioanal. Nucl. Chem. 2024, 333 (4), 2207–2220. 10.1007/s10967-024-09416-7.

[ref8] Day G. The Immobilisation of Caesium and Strontium from Nuclear Waste Captured by IONSIV; Ph.D. thesis; University of Birmingham, 2018.

[ref9] Chen T.-Y. Immobilisation of Caesium from Crystalline Silicotitanate by Hot Isostatic Pressing; Ph.D. thesis; University of Birmingham, 2012.

[ref10] Nyman M. D.; Nenoff T. M.; Headley T. J. Characterization of UOP IONSIV IE-911; Sandia National Lab.(SNL-NM)NM (United States): Albuquerque, 2001.

[ref11] National Research Council Alternatives for High-Level Waste Salt Processing at the Savannah River Site; National Academies Press, 2000.

[ref12] UOP A Honeywell Company. UOP IONSIVTM Ion Exchangers: a Superior Nuclear Waste Remediation Product; UOP A Honeywell Company: USA, 2012. UOP5649a.

[ref13] Panikorovskii T. L.; Kalashnikova G. O.; Nikolaev A. I.; Perovskiy I. A.; Bazai A. V.; Yakovenchuk V. N.; Bocharov V. N.; Kabanova N. A.; Krivovichev S. V. Ion-Exchange-Induced Transformation and Mechanism of Cooperative Crystal Chemical Adaptation in Sitinakite: Theoretical and Experimental Study. Minerals 2022, 12 (2), 248 10.3390/min12020248.

[ref14] Kesraoui-Ouki S.; Cheeseman C. R.; Perry R. Natural Zeolite Utilisation in Pollution Control: A Review of Applications to Metals’ Effluents. J. Chem. Technol. Biotechnol. 1994, 59 (2), 121–126. 10.1002/jctb.280590202.

[ref15] Celestian A. J.; Kubicki J. D.; Hanson J.; Clearfield A.; Parise J. B. The Mechanism Responsible for Extraordinary Cs Ion Selectivity in Crystalline Silicotitanate. J. Am. Chem. Soc. 2008, 130 (35), 11689–11694. 10.1021/ja801134a. 18683931

[ref16] Zhao X.; Meng Q.; Chen G.; Wu Z.; Sun G.; Yu G.; Sheng L.; Weng H.; Lin M. An Acid-resistant Magnetic Nb-Substituted Crystalline Silicotitanate for Selective Separation of Strontium and/or Cesium Ions from Aqueous Solution. Chem. Eng. J. 2018, 352, 133–142. 10.1016/j.cej.2018.06.175.

[ref17] Pease L. F.; Fiskum S. K.; Colburn H. A.; Schonewill P. P. Cesium Ion Exchange with Crystalline Silicotitanate Literature Review. Pacific Northwest National Laboratory; 2019 PNNL-28343, Rev. 0.

[ref18] Emelity L. A. Operation And Control Of Ion-Exchange Processes For Treatment Of Radioactive Wastes; International Atomic Energy Agency, 1967.

[ref19] International Atomic Energy Agency Treatment of Low-and Intermediate-Level Liquid Radioactive Wastes; International Atomic Energy Agency, 1984.

[ref20] Hooper W. Use of Inorganic Sorbents for Treatment of Liquid Radioactive Waste and Backfill of Underground Repositories IAEA Vienna 1992

[ref21] Gardner L. J.; Walling S. A.; Corkhill C. L.; Hyatt N. C. Thermal Treatment of Cs-Exchanged Chabazite by Hot Isostatic Pressing to Support Decommissioning of Fukushima Daiichi Nuclear Power Plant. J. Hazard. Mater. 2021, 413, 125250 10.1016/j.jhazmat.2021.125250. 33581672

[ref22] Krall L. M.; Macfarlane A. M.; Ewing R. C. Nuclear Waste From Small Modular Reactors. Proc. Natl. Acad. Sci. U. S. A. 2022, 119 (23), e2111833119 10.1073/pnas.2111833119. 35639689 PMC9191363

[ref23] Jenni A.; Hyatt N. Encapsulation of Caesium-loaded Ionsiv in Cement. Cem. Concr. Res. 2010, 40 (8), 1271–1277. 10.1016/j.cemconres.2009.10.015.

[ref24] Bahmanrokh G.; Whitelock E.; Dayal P.; Farzana R.; Koshy P.; Gregg D. J. Candidate Glass–Ceramic Wasteforms for the Immobilisation of Cs-loaded IONSIV® Wastes: A Scoping Study. MRS Adv. 2024, 9, 420–425. 10.1557/s43580-024-00830-3.

[ref25] Ojovan M. I.; Yudintsev S. V. Glass, ceramic, and glass-crystalline matrices for HLW immobilisation. Open Ceramics 2023, 14, 100355 10.1016/j.oceram.2023.100355.

[ref26] Chen T.-Y.; Maddrell E. R.; Hyatt N. C.; Gandy A. S.; Stennett M. C.; Hriljac J. A. Transformation of Cs-IONSIV® into a Ceramic Wasteform by Hot Isostatic Pressing. J. Nucl. Mater. 2018, 498, 33–43. 10.1016/j.jnucmat.2017.10.011.

[ref27] Chen T.-Y.; Hriljac J. A.; Gandy A. S.; Stennett M. C.; Hyatt N. C.; Maddrell E. R. Thermal Conversion of Cs-exchanged IONSIV IE-911 into a Novel Caesium Ceramic Wasteform by Hot Isostatic Pressing. MRS Online Proc. Libr. 2012, 1518, 67–72. 10.1557/opl.2013.202.

[ref28] Gregg D. J.; Farzana R.; Dayal P.; Holmes R.; Triani G. Synroc Technology: Perspectives and Current Status. J. Am. Ceram. Soc. 2020, 103 (10), 5424–5441. 10.1111/jace.17322.

[ref29] Konings R.; Stoller R. E. Comprehensive Nuclear Materials; Elsevier, 2020.

[ref30] Walker D. Cesium Sorption/Desorption Experiments with IONSIV (R) IE-911 in Radioactive Waste; Westinghouse Savannah River Company: Aiken, SC (United States), 2001.

[ref31] Prazska M.; Blazsekova M.; Green A.; Tuxworth A.; Howells R.; Invernizzi D. Comparison Between Cement and Geopolymers: Progress of Waste Encapsulation Using Geopolymers in the UK-21207. In WM2021 Conference, March 7 - 12, 2021, Phoenix, Arizona, USA.

[ref32] Andrews M. K.; Harbour J. R. Effect of CST Ion Exchange Loading on the Volume of Glass Produced During the Vitrification Demonstration at SRTC; Westinghouse Savannah River Company, 1996.

[ref33] Andrews M.; Workman P. Glass Formulation Development and Testing for the Vitrification of DWPF HLW Sludge Coupled with Crystalline Silicotitanate; CST, 1997.

[ref34] Kot W. K.; Pegg I. L.; Brandys M.; Penafiel M. Final Report: Vitrification of Inorganic Ion-Exchange Mediapp, VSL-16R3710–1; U.S. Department of Energy Office of Scientific and Technical Information, 2018

[ref35] Thorpe C. L.; Neeway J. J.; Pearce C. I.; Hand R. J.; Fisher A. J.; Walling S. A.; Hyatt N. C.; Kruger A. A.; Schweiger M.; Kosson D. S.; et al. Forty Years of Durability Assessment of Nuclear Waste Glass by Standard Methods. NPJ. Mater. Degrad. 2021, 5 (1), 61 10.1038/s41529-021-00210-4.

[ref36] Jantzen C.; Bibler N.; Beam D.; Crawford C.; Pickett M. Characterization of the Defense Waste Processing Facility (DWPF) Environmental Assessment (EA) Glass Standard Reference Material; Westinghouse Savannah River Co., 1993.

[ref37] Kvashnina K.; Claret F.; Clavier N.; Levitskaia T. G.; Wainwright H.; Yao T. Long-term, sustainable solutions to radioactive waste management. Sci. Rep. 2024, 14 (1), 5907 10.1038/s41598-024-55911-y. 38467714 PMC10928205

[ref38] Lutze W. Silicate Glasses. In Radioactive Waste Forms for the Future, Lutze W.; Ewing R. C., Eds.; Elsevier Science Pub. Co., Inc., 1988; pp. 3–159.

[ref39] Donald I.; Metcalfe B.; Taylor R. J. The Immobilization of High Level Radioactive Wastes using Ceramics and Glasses. J. Mater. Sci. 1997, 32 (22), 5851–5887. 10.1023/A:1018646507438.

[ref40] Lee W.; Ojovan M.; Stennett M.; Hyatt N. Immobilisation of Radioactive Waste in Glasses, Glass Composite Materials and Ceramics. Adv. Appl. Ceram. 2006, 105 (1), 3–12. 10.1179/174367606X81669.

[ref41] Carter M. L.; Gillen A. L.; Olufson K.; Vance E. R. HIPed Tailored Hollandite Waste Forms for the Immobilization of Radioactive Cs and Sr. J. Am. Ceram. Soc. 2009, 92 (5), 1112–1117. 10.1111/j.1551-2916.2009.03021.x.

[ref42] Zhao M.; Birkner N.; Schaeperkoetter J.; Koch R. J.; Russell P.; Misture S. T.; Besmann T.; Amoroso J.; Brinkman K. S. Durable Cr-Substituted (Ba, Cs)_1.33_(Cr, Ti)_8_O_16_ Hollandite Waste Forms with High Cs Loading. J. Am. Ceram. Soc. 2022, 105 (6), 4564–4576. 10.1111/jace.18419.

[ref43] Fang Z.; Xu X.; Yang X.; Xie H.; Zhao X.; Wang B.; Zhao D.; Yang Y. Structural Stability and Aqueous Durability of Cs Incorporation into BaAl_2_Ti_6_O_16_ Hollandite. J. Nucl. Mater. 2022, 565, 153716 10.1016/j.jnucmat.2022.153716.

[ref44] Carter M. L.; Vance E. R.; Mitchell D. R. G.; Hanna J. V.; Zhang Z.; Loi E. Fabrication, characterization, and leach testing of hollandite, (Ba,Cs)(Al,Ti)_2_Ti_6_O_16_. J. Mater. Res. 2002, 17 (10), 2578–2589. 10.1557/JMR.2002.0374.

[ref45] Papynov E.; Shichalin O.; Buravlev I. Y.; Belov A.; Fedorets A.; Ivanets A.; Tananaev I. Preparation of Pollucite Ceramic Matrices as ^137^Cs Ionizing Radiation Source by Spark Plasma Sintering. Ceram. Int. 2024, 50 (2), 2759–2771. 10.1016/j.ceramint.2023.10.341.

[ref46] Jing Z.; Hao W.; He X.; Fan J.; Zhang Y.; Miao J.; Jin F. A Novel Hydrothermal Method to Convert Incineration Ash into Pollucite for the Immobilization of a Simulant Radioactive Cesium. J. Hazard. Mater. 2016, 306, 220–229. 10.1016/j.jhazmat.2015.12.024. 26736173

[ref47] Omerašević M.; Matović L.; Ružić J.; Golubović Ž.; Jovanović U.; Mentus S.; Dondur V. Safe Trapping of Cesium into Pollucite Structure by Hot-Pressing Method. J. Nucl. Mater. 2016, 474, 35–44. 10.1016/j.jnucmat.2016.03.006.

[ref48] Nomura N.; Kikawada Y.; Oi T. Immobilization of Cesium by Zirconium Phosphate. J. Radioanal. Nucl. Chem. 2015, 304, 683–691. 10.1007/s10967-014-3853-z.

[ref49] Wang J.; Wei Y.; Wang J.; Zhang X.; Wang Y.; Li N. Simultaneous Immobilization of Radionuclides Sr and Cs by Sodium Zirconium Phosphate Type Ceramics and its Chemical Durability. Ceram. Int. 2022, 48 (9), 12772–12778. 10.1016/j.ceramint.2022.01.147.

[ref50] Roy R.; Vance E.; Alamo J. [NZP], A New Radiophase for Ceramic Nuclear Waste Forms. Mater. Res. Bull. 1982, 17 (5), 585–589. 10.1016/0025-5408(82)90040-X.

[ref51] Senamaud N.; Bernache-Assollant D.; Carpena J.; Fialin M. Cesium Incorporation into Phosphate Silicate Apatites. Phosphorus Res. Bull. 1999, 10, 353–358. 10.3363/prb1992.10.0_353.

[ref52] McCloy J. S.; Goel A. Glass-Ceramics for Nuclear-Waste Immobilization. MRM Bull. 2017, 42 (3), 233–240. 10.1557/mrs.2017.8.

[ref53] Zhang Y.; Kong L.; Ionescu M.; Gregg D. J. Current Advances on Titanate Glass-Ceramic Composite Materials as Waste forms for Actinide Immobilization: A Technical Review. J. Eur. Ceram. Soc. 2022, 42 (5), 1852–1876. 10.1016/j.jeurceramsoc.2021.12.077.

[ref54] Beger R. M. The Crystal Structure and Chemical Composition of Pollucite. Z. Fur Krist. -Cryst. Mater. 1969, 129 (1–6), 280–302. 10.1524/zkri.1969.129.16.280.

[ref55] Strachan D. M.; Schulz W. W. Characterization of Pollucite as a Material for the Long Term Storage of Cesium-137; IAEA, 1977.

[ref56] Orlova A. I.; Ojovan M. I. Ceramic Mineral Wasteforms for Nuclear Waste Immobilization. Materials 2019, 12 (16), 2638 10.3390/ma12162638. 31430956 PMC6719191

[ref57] Bell J. L.; Driemeyer P. E.; Kriven W. M. Formation of Ceramics from Metakaolin-Based Geopolymers: Part I—Cs-Based Geopolymer. J. Am. Ceram. Soc. 2009, 92 (1), 1–8. 10.1111/j.1551-2916.2008.02790.x.

[ref58] Mann N. R.; Todd T. A. Removal of Cesium from Acidic Radioactive Tank Waste by using Ionsiv IE-911. Sep. Sci. Technol. 2005, 39 (10), 2351–2371. 10.1081/SS-120039321.

[ref59] Juoi J.; Ojovan M.; Lee W. Microstructure and Leaching Durability of Glass Composite Wasteforms for Spent Clinoptilolite Immobilisation. J. Nucl. Mater. 2008, 372 (2–3), 358–366. 10.1016/j.jnucmat.2007.04.047.

[ref60] Thompson P.; Cox D.; Hastings J. Rietveld Refinement of Debye–Scherrer Synchrotron X-ray Data from Al_2_O_3_. J. Appl. Crystallogr. 1987, 20 (2), 79–83. 10.1107/S0021889887087090.

[ref61] ISO ISO18754:2020Fine Ceramics (Advanced Ceramics, Advanced Technical Ceramics) — Determination of Density and Apparent Porosity; ISO, 2016.

[ref62] ASTM C1285: standard Test Methods for Determining Chemical Durability of Nuclear, Hazardous, and Mixed Waste Glasses and Multiphase Glass Ceramics: The Product Consistency Test (PCT); ASTM, 2021.

[ref63] Bachina A.; Almjasheva O. V.; Danilovich D. P.; Popkov V. I. Synthesis, Crystal Structure, and Thermophysical Properties of ZrTiO_4_ Nanoceramics. Russ. J. Phys. Chem. 2021, 95 (8), 1529–1536. 10.1134/S0036024421080057.

[ref64] Motina A. G.; Pazukhin V. A.; Lainer A. I.; Kolenkova M. A. Sublimation of Cesium Oxide from Pollucite Caked With Lime in Vacuum. Zhur Priklad, Khim., 1962, 35.

[ref65] Chen S.; Guo J.-F.; Xu B.; Sun X.-W. Sintering of Metakaolin-based Na-Pollucite Ceramics and Their Immobilization of Cs. Ann. Nucl. Energy 2020, 145, 107595 10.1016/j.anucene.2020.107595.

[ref66] Gregg D. J.; Vance E. R.; Dayal P.; Farzana R.; Aly Z.; Holmes R.; Triani G. Hot Isostatically Pressed (HIPed) Fluorite Glass-Ceramic Wasteforms for Fluoride Molten Salt Wastes. J. Am. Ceram. Soc. 2020, 103 (10), 5454–5469. 10.1111/jace.17293.

[ref67] Kuisma-Kursula P. Accuracy, precision and detection limits of SEM–WDS, SEM–EDS and PIXE in the Multi-Elemental Analysis of Medieval Glass. X-Ray Spectrom. 2000, 29 (1), 111–118. 10.1002/(SICI)1097-4539(200001/02)29:1<111::AID-XRS408>3.0.CO;2-W.

[ref68] Kot W. K.; Pegg I. L.; Brandys M.; Penafiel M. Vitrification of Inorganic Ion-Exchange Media, VSL-16R3710–1 (No. ORP-61830); Hanford Site (HNF); WA (United States): Richland, 2018.

[ref69] Grote R.; Hong T.; Shuller-Nickles L.; Amoroso J.; Tang M.; Brinkman K. Radiation Tolerant Ceramics for Nuclear Waste Immobilization: Structure and Stability of Cesium Containing Hollandite of the Form (Ba, Cs)_1.33_(Zn, Ti)_8_O_16_ and (Ba, Cs)_1.33_(Ga, Ti)_8_O_16_. J. Nucl. Mater. 2019, 518, 166–176. 10.1016/j.jnucmat.2019.03.005.

[ref70] Vance E. R.; Chavara D. T.; Gregg D. J. Synroc Development—Past and Present Applications. MRS Energy Sustainability 2017, 4, 8 10.1557/mre.2017.9.

[ref71] Kim G.-Y.; Shin S.-S.; Lee B.; Choi J.-H.; Kang H. W.; Pyo J.-Y.; Yang J. H.; Park H.-S.; Lee K. R. Characteristics of Cs Pollucite Synthesized at Various Cs Loadings for Immobilization of Radioactive Cs. J. Nucl. Mater. 2024, 588, 154781 10.1016/j.jnucmat.2023.154781.

[ref72] Ewing R.; Lutze W.; Weber W. J. Zircon: A Host-Phase for the Disposal of Weapons Plutonium. J. Mater. Res. 1995, 10, 243–246. 10.1557/JMR.1995.0243.

[ref73] Shannon R. D. Acta Crystallographica Section A: Crystal Physics, Diffraction. Theor. Gen. Crystallogr. A 1976, 32, 751–767.

[ref74] Gera F. The Classification of Radioactive Wastes. Health Phys. 1974, 27 (1), 113–121. 10.1097/00004032-197407000-00015. 4430609

[ref75] Yanagisawa K.; Nishioka M.; Yamasaki N. Immobilization of Cesium into Pollucite Structure by Hydrothermal Hot-Pressing. J. Nucl. Sci. Technol. 1987, 24 (1), 51–60. 10.1080/18811248.1987.9735774.

[ref76] Curi R. F.; Luca V. In-column Immobilization of Cs-Saturated Crystalline Silicotitanates using Phenolic Resins. Environ. Sci. Pollut. Res. 2018, 25, 6850–6858. 10.1007/s11356-017-1019-6. 29270894

[ref77] Andrews M. Glass Formation Development and Testing for the Vitrification of Cesium-Loaded Crystalline Silicotitanate (CST); Savannah River Site (SRS): Aiken, SC (United States), 1997.

