# Supplementary material for: Pollucite Ceramics and Glass-Ceramics as Advanced Wasteforms for the Immobilization of Cs-Loaded IONSIV Wastes
Source: Environ Sci Technol. 2025 Apr 15;59(16):7948–59. doi: 10.1021/acs.est.5c00266 (PMC12044701; doi:10.1021/acs.est.5c00266)
Supplement: Supplementary file 1 — es5c00266_si_001.pdf [file es5c00266_si_001.pdf]

# Supporting Information

# Pollucite Ceramics and Glass-Ceramics as Advanced Wasteforms for the Immobilization of Cs-loaded IONSIV Wastes

*Ghazaleh Bahmanrokh[1*], Edward Whitelock[2], Pranesh Dayal[1], Robert D. Aughterson[1], Anton Peristyy[1], Phillip Sutton[1], Rifat Farzana[1], Joel L. Abraham[1,2], Jess Degeling[1,2], Michael Page[1], Charles C. Sorrell[2], Pramod Koshy[2], and Daniel J. Gregg[1]*

Number of pages = 13

Number of figures = 9

Number of tables = 5

Number of notes = 2

## 2. EXPERIMENTAL PROCEDURE

**Table S1**. $D_{10}$, $D_{50}$, and $D_{90}$ results for calcined and milled powders for compositions CsL-C, CsL-GC1 and CsL-GC2. All particle size ranges were analyzed using laser diffraction (Mastersizer)[1].

| Sample Identification | Particle Size[1] (μm) | | |
|---|---|---|---|
| | **$D_{10}$** | **$D_{50}$** | **$D_{90}$** |
| CsL-C | 0.69 | 1.97 | 6.50 |
| CsL-GC1 | 0.51 | 0.97 | 4.1 |
| CsL-GC2 | 0.61 | 1.50 | 5.5 |

[1] Particle sizes were measured using laser diffraction with a Malvern PANalytical Mastersizer 3000 and the $D_{50}$ (volume weighted mean) results were recorded.

## 3. RESULTS AND DISCUSSION

**Table S2**. Targeted elemental wt.% and measured elemental wt.% from XRF for CsL-C and CsL-GCs wasteforms.

| **Element** | **Concentration in Wasteform, % w/w** | | | | | |
|---|---|---|---|---|---|---|
| | **CsL-GC1** | | **CsL-GC2** | | **CsL-C** | |
| | Targeted | Measured | Targeted | Measured | Targeted | Measured |
| **Al** | 2.831 | 2.45 ± 0.15 | 3.451 | 3.03 ± 0.18 | 3.24 | 11.99 ± 0.60 |
| **B** | 0.071 | NR | 0.497 | NR | NP | NP |
| **Cs** | 13.092 | 12.7 ± 0.6 | 11.111 | 11.3 ± 0.6 | 13.29 | 11.88 ± 0.59 |
| **Fe** | 7.932 | 7.73 ± 0.39 | 6.729 | 6.74 ± 0.34 | 8.05 | 7.13 ± 0.36 |

| | | | | | | |
|---|---|---|---|---|---|---|
| **Na** | 0.697 | 0.62 ± 0.12 | 2.381 | 2.03 ± 0.20 | 0.41 | 0.35 ± 0.07 |
| **Nb** | 13.198 | 11.7 ± 0.35 | 11.199 | 10.1 ± 0.30 | 13.40 | 11.09 ± 0.33 |
| **Si** | 6.712 | 6.54 ± 0.33 | 10.064 | 10.1 ± 0.50 | 6.08 | 4.98 ± 0.25 |
| **Ti** | 12.680 | 13.8 ± 0.41 | 10.761 | 11.6 ± 0.35 | 12.87 | 9.49 ± 0.28 |
| **Zr** | 10.261 | 11.5 ± 0.35 | 8.706 | 9.42 ± 0.28 | 10.42 | 8.61 ± 0.43 |

NR = not reported

NP = not presented

**Table S3**. Composition (oxide wt.%) of UL and CsL IONSIV samples as determined by ICP-MS and XRF.

| **Material** | **UL IONSIV** | | | **CsL IONSIV** | | | |
|---|---|---|---|---|---|---|---|
| **Thermal Treatment** | **None** | **None** | **1100 ˚C** | **None** | **600 ˚C** | **800 ˚C** | **1100 ˚C** |
| **Analysis Method** | **ICP-MS** | **XRF[1]** | | | | | |
| $Na_2O$ | 4.2 | 4.3 | 4.5 | 0.6 | 0.5 | 0.7 | 0.5 |
| $Nb_2O_5$ | 23.4 | 28.0 | 25.4 | 24.4 | 23.6 | 23.3 | 23.2 |
| $SiO_2$ | 19.9 | 17.9 | 18.6 | 15.3 | 15.8 | 15.8 | 15.8 |
| $TiO_2$ | 34.3 | 30.6 | 31.6 | 26.1 | 26.2 | 26.1 | 26.0 |
| $ZrO_2$ | 18.0 | 19.2 | 20.0 | 16.7 | 17.2 | 17.1 | 17.1 |
| $Cs_2O$ | - | - | - | 16.9 | 16.8 | 17.1 | 17.3 |
| $SO_3$ [(2)] | - | - | - | 2.2 | 2.2 | 1.9 | 0.5 |

1 All results exclude water content.

2 A small quantity of sulphur was detected due to the Cs-loading method employed and this was significantly reduced by thermal treatment at temperatures ≥ 1100 ˚C.

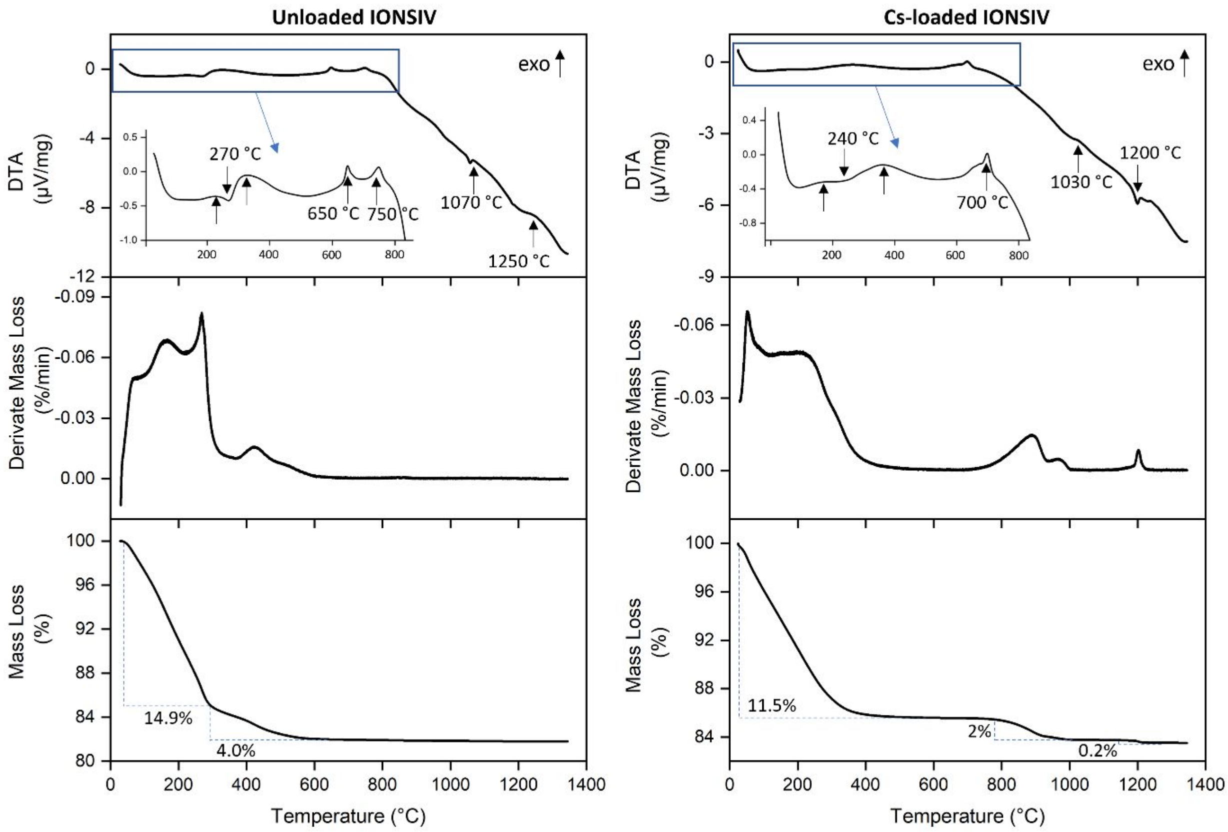


**Figure S1.** TGA, DTG, and DTA data for the UL and CsL IONSIV samples under Ar gas.

The mass loss between 750˚ – 1000 ˚C observed for the CsL sample may indicate volatilization of S containing species [1, 2].

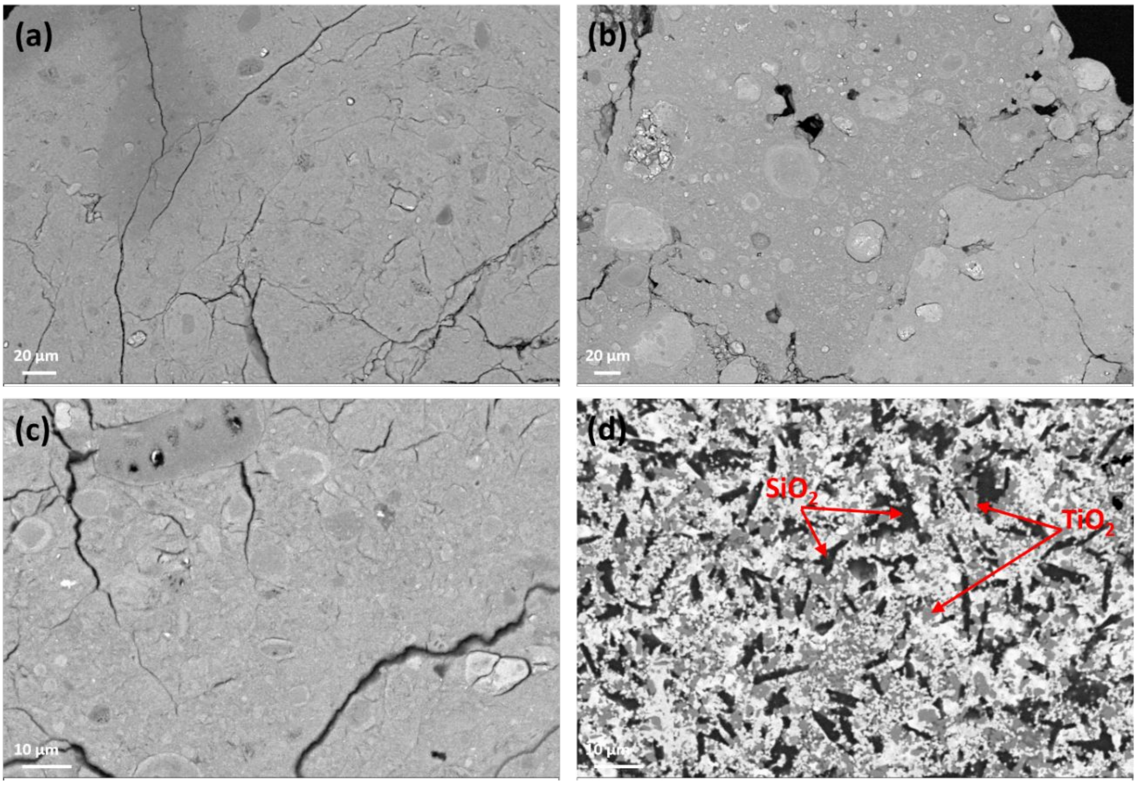


**Figure S2**. SEM images of UL IONSIV. (a) before sintering and (b) after sintering at 600 ˚C, (c) 800 ˚C, and (d) 1100 ˚C for 1 h in air.

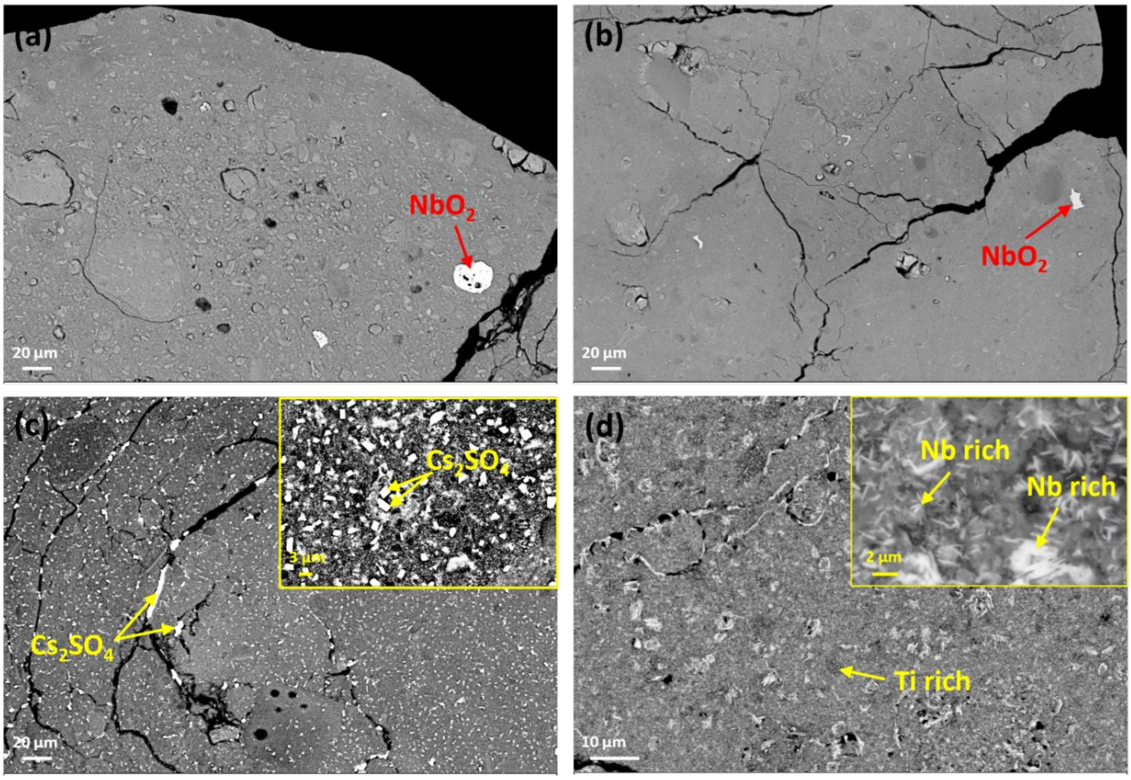


**Figure S3**. SEM images of CsL IONSIV. (a) before sintering and (b) after sintering at 600 ˚C, (c) 800 ˚C, and (d) 1100 ˚C for 1 h in air.

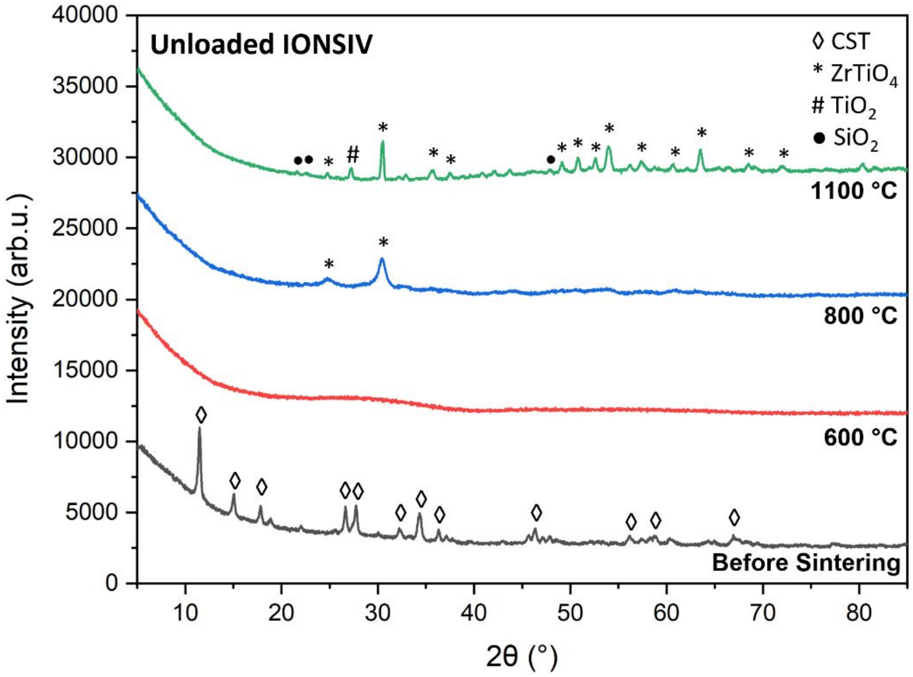

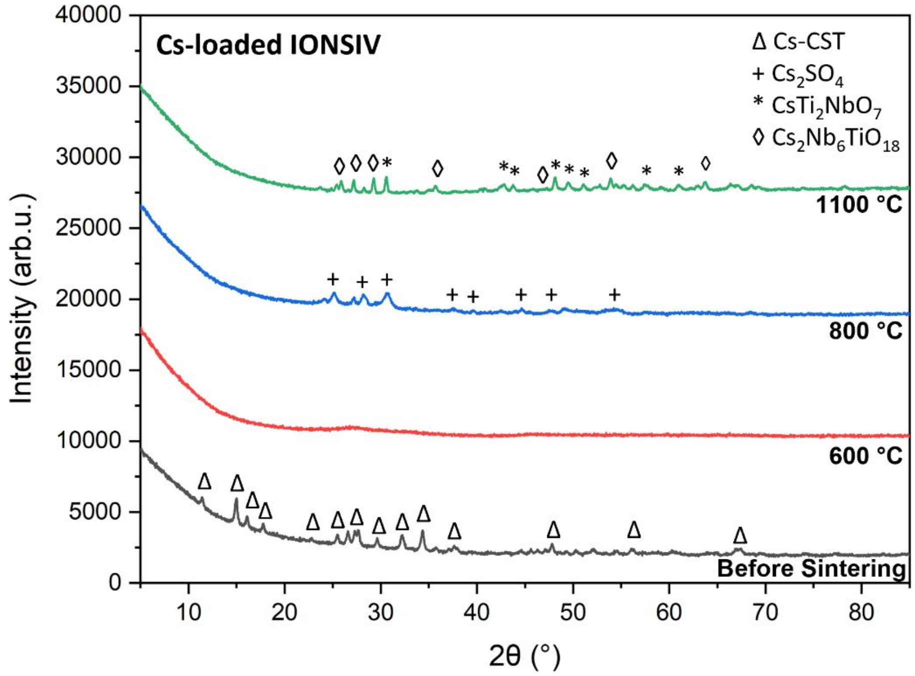


**Figure S4**. XRD patterns of UL and CsL IONSIV samples before sintering and after sintering at 600 ˚C, 800 ˚C, and 1100 ˚C in air for 1 h.

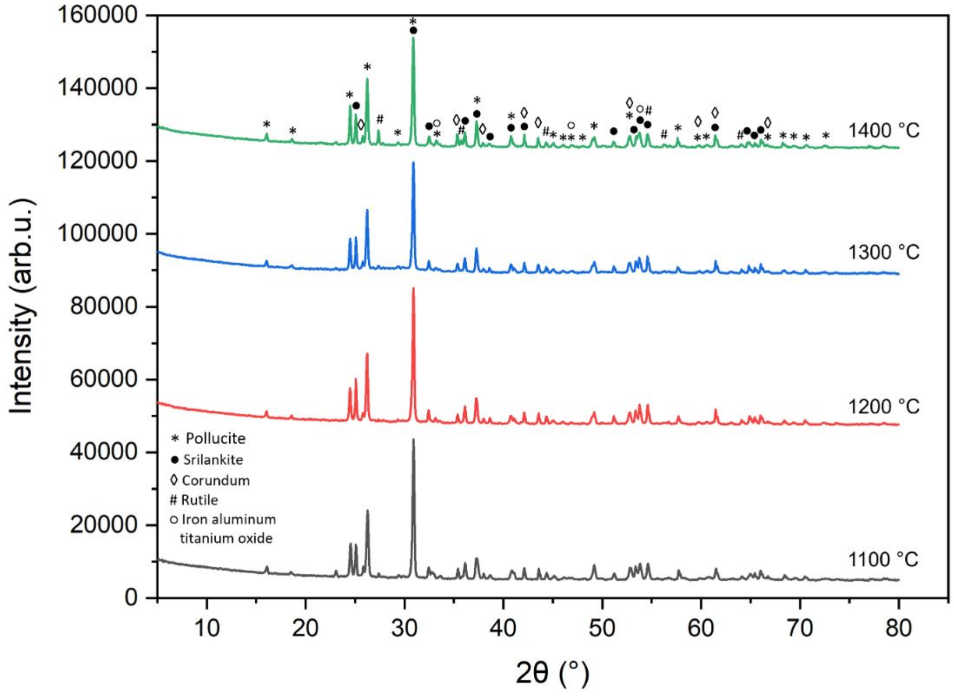

**Figure S5.** XRD patterns for CsL-C wasteform sintered at 1100° – 1400 °C in air for 6 h. The main peaks in the pattern are pollucite and srilankite. Very weak peaks in the pattern are related to corundum, rutile, and iron aluminum titanate.

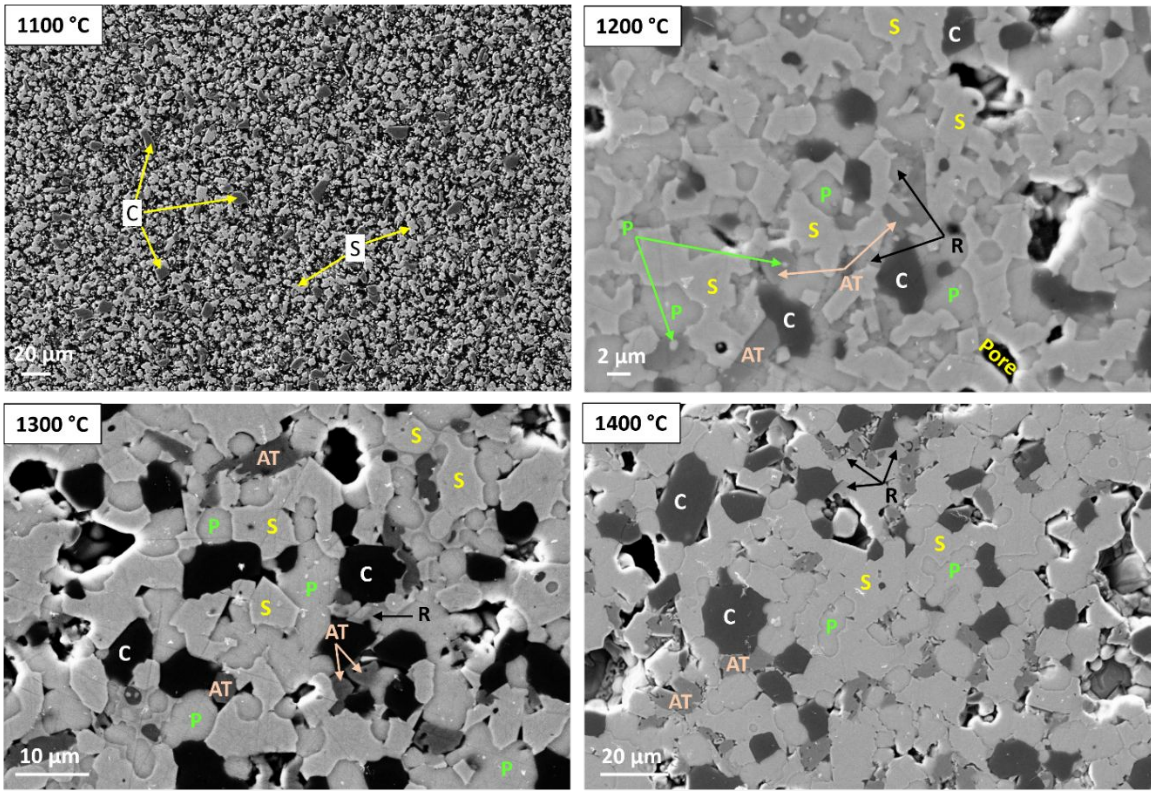


**Figure S6.** Secondary SEM images for CsL-C wasteform sintered at 1100° – 1400 °C in air for 6 h. The phases are labelled as (P) pollucite, (S) srilankite, (R) rutile, (C) corundum, and (AT) aluminium titanate.

**Table S4.** Summary of the Cs-partitioning content calculated from EDS-SEM data and quantitative phase analysis data from Rietveld refinement.

| Sample Identifier | CsL-GC1 | CsL-GC2 | UL-VAL | CsL-VAL |
|---|---|---|---|---|
| Target glass Content | 3 | 20 | 20 | 20 |

| (wt.%) | | | | |
|---|---|---|---|---|
| | | Cs Partitioning (%)[1] | | |
| **Ceramic** | 99 | 93 | - | 91 (93[2]) |
| **Glass** | 1 | 7 | - | 9 (7[2]) |
| **Phase** | | **Phase Quantity (%)** | | |
| Pollucite | 35.9 | 34.1 | -- | 29.0 |
| Srilankite | 47.6 | 45.0 | 47.0 | 41.2 |
| Rutile | 16.4 | 20.8 | 51.7 | 29.9 |
| Zircon | -- | -- | 1.3 | -- |
| | | *Agreement Indices* | | |
| *GOF* (*goodness of fit*) | 6.2 | 4.7 | 10.2 | 5.7 |
| *Rexp* (R expected) | 1.2 | 1.3 | 4.0 | 3.9 |
| *Rwp* (Weighted R profile) | 3.1 | 2.9 | 11.3 | 9.4 |

[1] SEM-EDS results for Cs concentration in the glass phase were used in combination with the target glass content (3 or 20 wt.%) to determine the degree of partitioning of Cs into the glass phase.

[2] TEM-EDS results for Cs concentration in the glass phase were used in combination with the target glass content (20 wt.%) to determine the degree of partitioning of Cs into the glass phase.

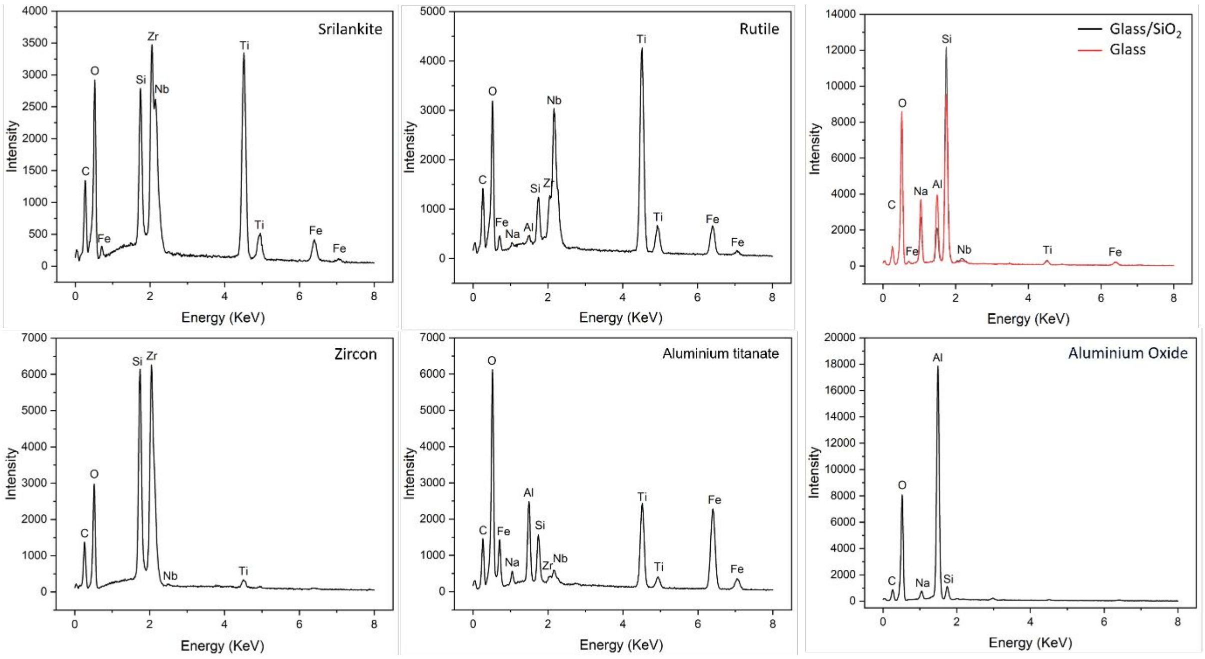


**Figure S7**. EDS patterns of phases in UL-VAL wasteform sintered at 1100 ˚C in air for 6 h.

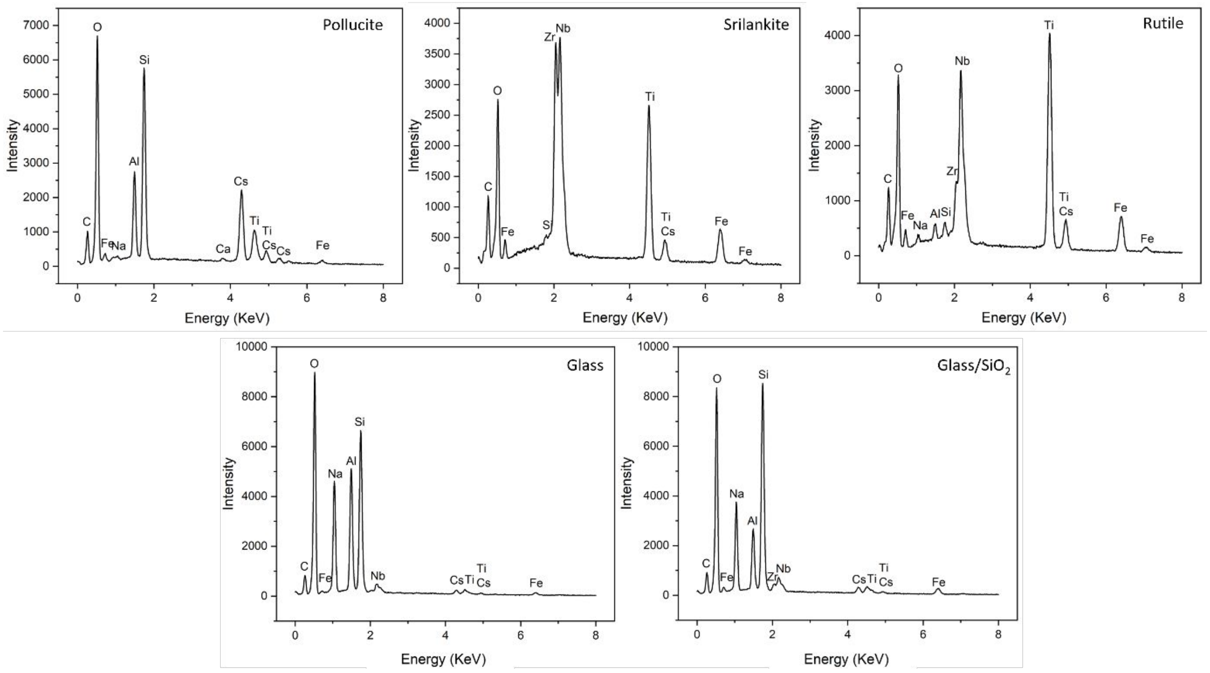


**Figure S8**. EDS patterns of phases in CsL-VAL wasteform sintered at 1100 ˚C in air for 6 h.

**Table S5**. Summary of the average composition of phases from the TEM-EDS analysis for UL-VAL, and CsL-VAL wasteforms.

| Phase | UL-VAL | CsL-VAL |
| --- | --- | --- |
| | **Composition from TEM** | |
| Pollucite | NP | $CsAlSi_2O_6$ [1] |
| Srilankite | $(Ti_{0.48}Zr_{0.23}Nb_{0.15}Fe_{0.14})O_2$ | $(Ti_{0.34}Zr_{0.20}Nb_{0.16}Fe_{0.15})O_2$ |
| Rutile | $(Ti_{0.55}Zr_{0.09}Nb_{0.18}Fe_{0.18})O_2$ | $(Ti_{0.56}Zr_{0.06}Nb_{0.19}Fe_{0.18})O_2$ |
| Glass | $Na_{1.1}Al_{0.6}B_{0.5}Si_2O_6$ [2] | $Na_{1.1}Al_{0.5}B_{0.5}Si_2O_6$ [2] |

NP = Phase not present.

[1] Also contains minor Na (~0.05 f.u.) and Ti (~0.05 f.u.) and Fe (~0.10).

[2] Assumed value for B as it not measurable using the TEM-EDS system employed. Also contains minor Cs (~0.07 f.u.), Ti (~0.1 f.u.), Fe (~0.1 f.u), Zr (~0.05) and Nb (0.1).

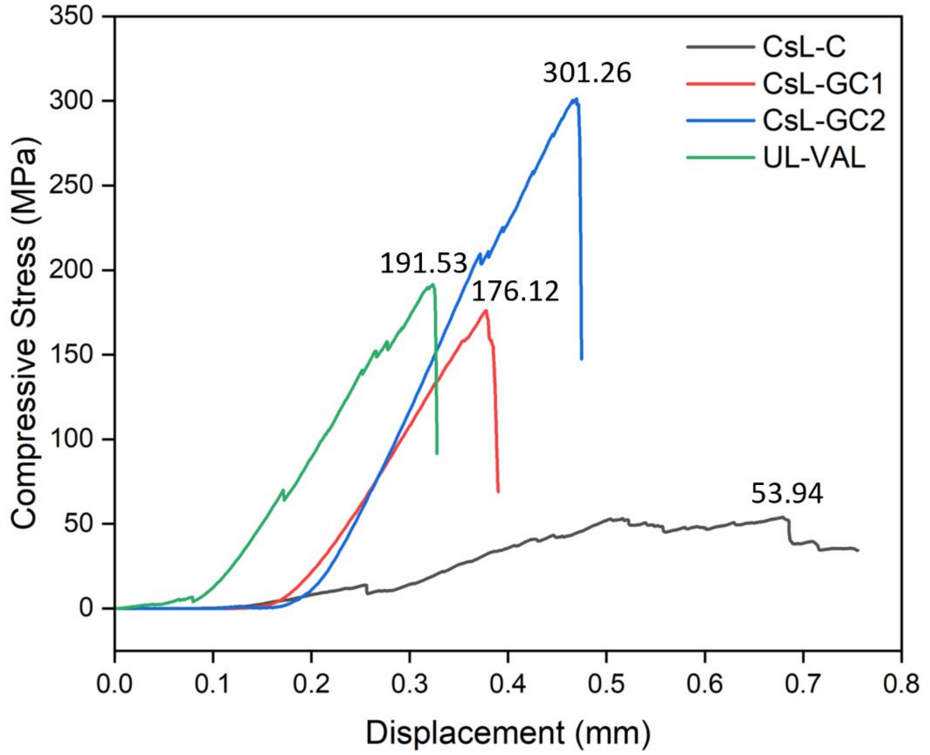


**Figure S9**. Mechanical durability with compressive strength values of CsL-C, CsL-GCs, and UL-VAL wasteforms sintered at 1100 ˚C in air for 6 h. Compression tests were conducted at a loading rate of 0.3 mm/min until failure, using a calibrated Instron 5967.

**Note 1: Cs-Partitioning:**

When comparing the individual CsL-GC samples, CsL-GC1 did show a higher degree of partitioning in ceramic phase relative to CsL-GC2; however, this was likely a result of the very low glass content for CsL-GC1. Though the Cs content for the glass phase (obtained from SEM-EDS data) in each of CsL-GC1 and CsL-GC2 was similar, the former had significantly less glass (phase abundance) than the latter (3 and 20 wt.%, respectively) and thus the total Cs inventory in the glass phase for CsL-GC1 was lower than that for CsL-GC2. By extension, it is anticipated that if additional glass was included in the design (e.g., 30 – 50 wt.%) the amount of Cs partitioned to the ceramic phase may be reduced further, unless the composition of the glass phase could be modified to hinder the incorporation of Cs. Given that Cs-137 is a major component of high-level radioactive waste, its behavior as a component of nuclear waste glass wasteforms is well attested in the literature [3-6], and thus optimization of the glass phase formulation to minimize Cs incorporation may be practicable.

**Note 2: List of assumptions for activity concentration, heat output and contact gamma dose:**

The heat output generated by the wasteform can be estimated using the following assumptions:

1. A theoretical wasteform package size of 5 L (e.g., a 5 L HIPed canister), with a density of 3.9 g/cm$^3$ (measured true density of CsL-GC2) translates to 19.5 kg of wasteform per wasteform package.
2. The IONSIV® waste loading within the wasteform design is 70 wt.%, meaning 13.7 kg of the wasteform package consists of IONSIV® waste.

3. The Cs-137 concentration in the IONSIV® waste is 0.002 %w/w (provided as an example for the current calculation), would equate to 0.272 g of Cs per wasteform package.
4. The Cs in the waste consists solely of the Cs-137 radionuclide. This corresponds to Cs-137 loading in the wasteform of 176 GBq/L, which is comparable to typical activity concentrations in waste classified as US class-C waste (4600 Ci/$m^3$, or 170 GBq/L).
5. The heat output for Cs-137 can be approximated as 0.42 W/g. This is based upon the conservative assumption that 100% of the γ-decay energy and 35% of the β-decay energy is deposited within the wasteform, while 65% of the β-decay energy is lost as antineutrino radiation [7].

Based on the above assumptions, one wasteform package would produce approximately 0.11 W of radiogenic heat. Given the specific activity of Cs-137 is 3.215 x $10^{12}$ Bq/g, the wasteform package would have an activity concentration of $4.5 \times 10^7$ Bq/g. Such a package will have an estimated gamma dose rate on contact of 3 Gy/h (Estimated using Microshield® Pro 13.10X software).

REFERENCES

(1) Pletser, D.; Ohashi, T.; Yoshii, Y.; Lee, W. Temperature Dependent Volatilisation Behaviour of Cs from two Commercial Adsorbents used at Fukushima Measured using Novel Experimental Apparatus. *Progress in Nuclear Energy* **2018**, *109*, 214-222.

(2) Munthali, M.; Johan, E.; Aono, H.; Matsue, N. $Cs^+$ and $Sr^{2+}$ Adsorption Selectivity of Zeolites in Relation to Radioactive Decontamination. *Journal of Asian ceramic societies* **2015**, *3* (3), 245-250.

(3) Stefanovsky, S. V.; Yudintsev, S. V.; Gieré, R.; Lumpkin, G. R. Nuclear Waste Forms. *Geological Society, London, Special Publications* **2004**, *236* (1), 37-63.

(4) Lutze, W. Silicate Glasses. In *Radioactive Waste Forms for the Future*, Lutze, W., Ewing, R. C. Eds.; 1988; pp 3-159.

(5) Donald, I.; Metcalfe, B.; Taylor, R. J. The Immobilization of High Level Radioactive Wastes using Ceramics and Glasses. *Journal of Materials Science* **1997**, *32* (22), 5851-5887.

(6) Stoch, P. Cs Containing Borosilicate Waste Glasses. *Optica Applicata* **2008**, *38* (1).

(7) Live Chart of Nuclides. IAEA - Nuclear Data Section. https://www-nds.iaea.org/relnsd/vcharthtml/VChartHTML.html (accessed).
